# Eastern Himalayan syntaxis formation controlled by slab flattening and crustal shear localization

**DOI:** 10.1038/s41561-026-02045-7

**Published:** 2026-07-20

**Authors:** Luuk van Agtmaal, Attila Balázs, Sean Willett, Dave May, Taras Gerya

**Affiliations:** 1 Geophysical Fluid Dynamics Group, Department of Earth and Planetary Sciences, ETH Zürich, Zürich, Switzerland; 2 Earth Surface Dynamics Group, Department of Earth and Planetary Sciences, ETH Zürich, Zürich, Switzerland; 3 Scripps Institution of Oceanography, University of California San Diego, La Jolla, CA USA

**Keywords:** Geology, Tectonics

## Abstract

Plate corners in collisional orogens are often characterized by sharply curved orogenic zones, that is, orogenic syntaxis zones, where tectonic mountain building and surface processes vigorously interact. The formation and evolution of syntaxis zones, which are particularly well expressed in the eastern and western terminations of the Himalayan orogen, remain debated. Here, based on 3D thermomechanical surface process numerical experiments, we propose that the formation of the Eastern Himalayan syntaxis is predominantly driven by flattening of the Indian slab and shear localization in the weak Eurasian crust. Our models indicate that a northward migrating syntaxis initially forms during collision with India when the middle to lower crust in the Eurasian plate is rheologically weak beneath a proto-plateau. This syntaxis becomes a barrier for the laterally expanding plateau. The corner of the collision zone is dismembered by the localization of crustal-scale strike–slip fault zones driven by slab flattening and differential plate advance. The middle to lower crust locally flows within a narrow channel from the plateau towards the east and south along the syntaxis. These links between slab dynamics and crustal shear localization drive the structural and topographic evolution of orogenic syntaxis.

## Abstract (web summary)

The Eastern Himalayan orogenic corner, or syntaxis, is largely formed by flattening of the Indian slab and shear localization in weak crust, which may affect development of the orogenic system, according to thermomechanical models.

## Main

**Fig. 1:**
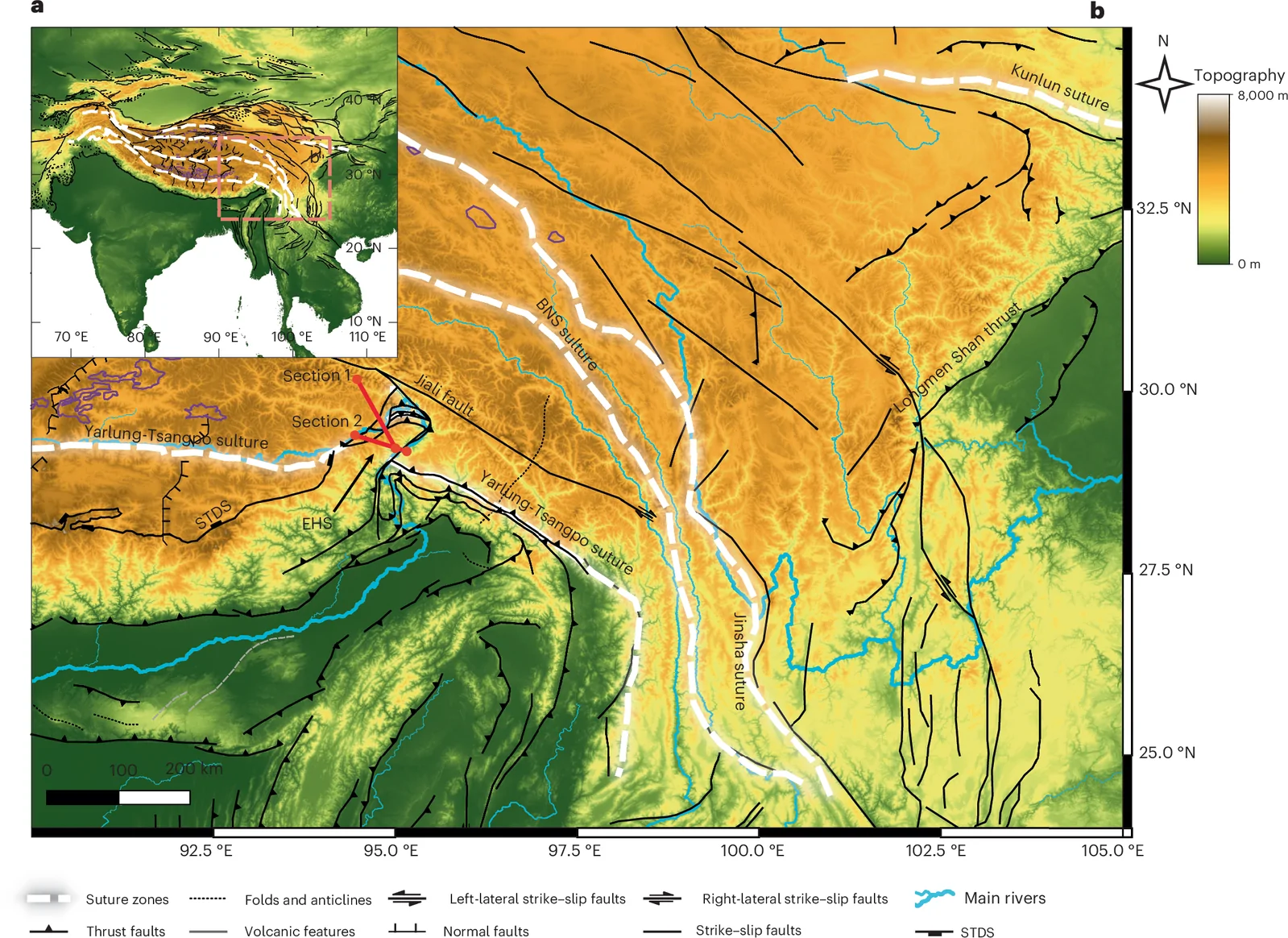
Tectonic map of the EHS and surrounding area. **a**,**b**, Topography map of the Himalayan–Tibetan orogenic system (**a**) with a close-up on the eastern corner (**b)**. The syntaxis is located in the eastern tip of the Himalayas. It consists of a plunging antiform with a pop-up structure in its core, as depicted in the two simplified geological cross sections in Extended Data Fig. 6. Map generated with QGIS with SRTM90 topographic data, rivers and regional structures from ref. ^39^ and fault zone traces around the EHS from ref. ^8^. IND: Indian Plate; EUR: Eurasian Plate; TB: Tarim Basin; SB: Sichuan Basin; CSSZ: Chongshan shear zone; GSZ: Gaoligong shear zone.

The Tibetan–Himalayan orogenic system is one of the most impressive features of plate tectonics and continental landscape evolution on Earth^1–3^. The orogenic system consists of the Himalayas and Tibetan Plateau and terminates towards the Hengduan Mountains of southeastern Asia (Fig. 1). The Tarim cratonic basin, the North China craton and the Sichuan cratonic basins confine the orogenic system in the north and east, respectively (Fig. 1a), and have acted as barriers to Cenozoic plateau expansion^3,4^.

Collisional zone corners host pronounced along-strike kinematic and dynamic variations, making them distinct from orogen interiors^5–7^. Such corners record spatio-temporal changes of the stress and thermal fields, deformation and uplift, which are sensitive proxies for tectonics–climate interactions^5^. Areas where the strike of the orogenic front abruptly changes at the corners of the collision zone are referred to as orogenic syntaxes^6,7^. How syntaxes form remains an open question^8^. There are currently two main ocean–continent syntaxes: the Olympic Mountains in the Cascadia subduction zone and the Saint Elias syntaxis in the Aleution–Alaskan subduction zone^9,10^. There are also two continent–continent syntaxes, that is, Nanga Parbat and Namche Barwa in the western and eastern terminations of the Himalayas, respectively^6–13^. Here we address the formation and evolution of the Eastern Himalayan syntaxis (EHS), which notably features rapid exhumation^14,15^ and intense erosion^5,16^. We focus on the large-scale tectonics of the EHS and not on the local, sharp curves of the Yarlung–Tsangpo suture zone (Fig. 1) that are also referred to as syntaxes^12^. The retreating subduction zones of SE Asia represents a free boundary, making the EHS an ideal site to study syntaxis formation and plateau growth processes^1–3^.

The mechanisms governing the formation of the EHS have remained enigmatic, including the potential role and duration of mid–lower crustal flow underneath the Tibetan Plateau and Hengduan Mountains. Ding et al.^8^ proposed two conceptual models for EHS formation: an exhumed crustal pop-up structure within the syntaxis that is part of a larger duplex and/or folded Indian crust indenting Tibetan crust. There is no consensus on whether the syntaxis influenced uplift and lateral expansion of the Himalayas and Tibetan Plateau. King et al.^17^ argued for northward migration of the syntaxis over the past 1 Myr indicating a shifting erosional centre. In contrast, the tectonic aneurysm model postulates that surface processes and tectonic uplift enhance each other, localizing a ‘bulls-eye’ area of erosion and uplift of weak, hot material^14^. Similar hypothesis combining curved subducting plate geometry and high erosion potential has been studied by numerical models^11,18^. Syntaxis formation has been modelled in 2D to reproduce the basin–antiform–basin geometry^19^ and the role of viscous heating^20^. Analogue models indicate that relative transversal flow over the indenter and continuous continental subduction enhance syntaxis curvature^12^. Three-dimensional studies have focused on the effects of lithosphere rheology and slab-induced mantle flow^21–25^. However, none of these studies modelled spontaneous and self-consistent syntaxis formation. Mid–lower crustal flow under Tibet is often referred as either erosion controlled, channelized exhumation of the Greater Himalaya Crystalline Complex^26^ or to lateral extrusion of thickened and weakened mid–lower crust from central to (south-)eastern Tibet^27,28^. This mid–lower crustal flow hypothesis was proposed to explain topographic growth in the eastern and southeastern Tibetan Plateau.^28^. The hypothesis is based on geophysical inferences of fluids and partially molten crust beneath the plateau^29^. This was supported by numerical modelling results. For example, channels formed readily in both 2D steady-state models with an S point (a stress singularity) and prescribed geometry^26^ and in 3D thin-viscous-sheet models with constant-viscosity layers^30,31^. However, 2D models cannot address the along-strike variations of channel flow. By definition, thin-viscous-sheet models cannot incorporate brittle crustal behaviour. Furthermore, latest studies of large thrusts in SE Tibet, such as the Muli thrust, and its 2D numerical models contradict the crustal channel flow theory^32^. Thus, channel flow remains enigmatic and controversial.

**Extended Data Fig. 1:**
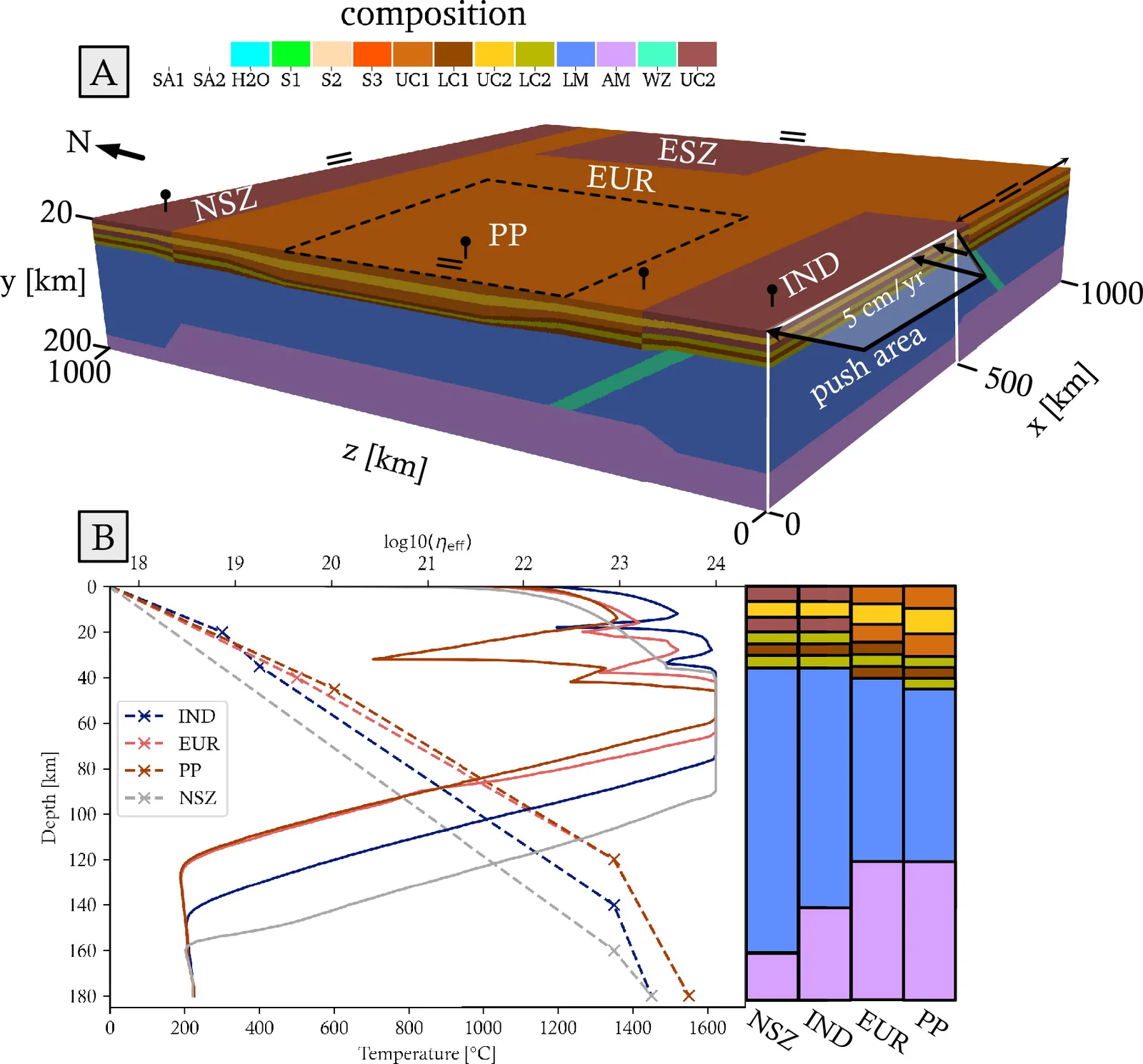
Reference model setup. Initial setup of the reference model showing the plate configuration and kinematic boundary conditions in panel **A**. Panel **B** depicts the rheological contrasts obtained by imposing different crustal thicknesses and initial geothermal profiles. Upper and lower crust have sublayers with otherwise identical properties (Extended Data Table 1). Pins in panel **A** indicate locations of effective viscosity profiles taken in an early timestep. The proto-plateau is the weakest unit due its thick, warm upper crust. IND indenting “Indian” plate; EUR “Eurasian” plate; PP - Proto-plateau, NSZ – Northern strong zone representing the Tarim basin; ESZ - Eastern Strong zone representing the Sichuan basin. SA – Sticky air; UC- Upper crust; LC – Lower crust; LM – Lithospheric mantle; AM – Asthenospheric mantle; WZ – Weak zone.

**Extended Data Fig. 2:**
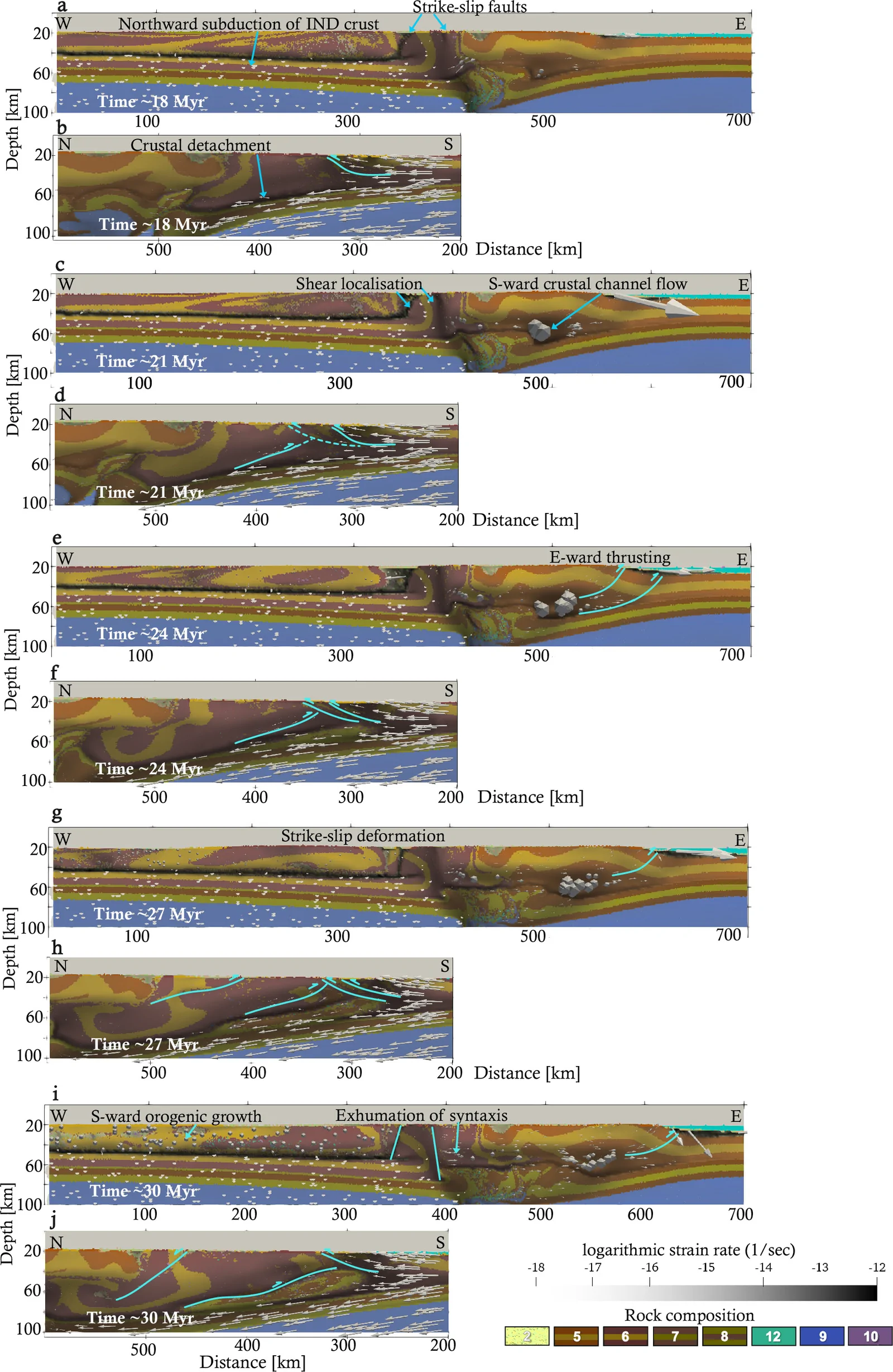
Active deformation in the syntaxis between 18-30 Myr. **a-j** Rock composition cross-sections oriented west-east and north-south, overlain by velocity arrows in white, logarithmic strain rate field in grey and highlighting main faults in blue.

**Extended Data Fig. 3:**
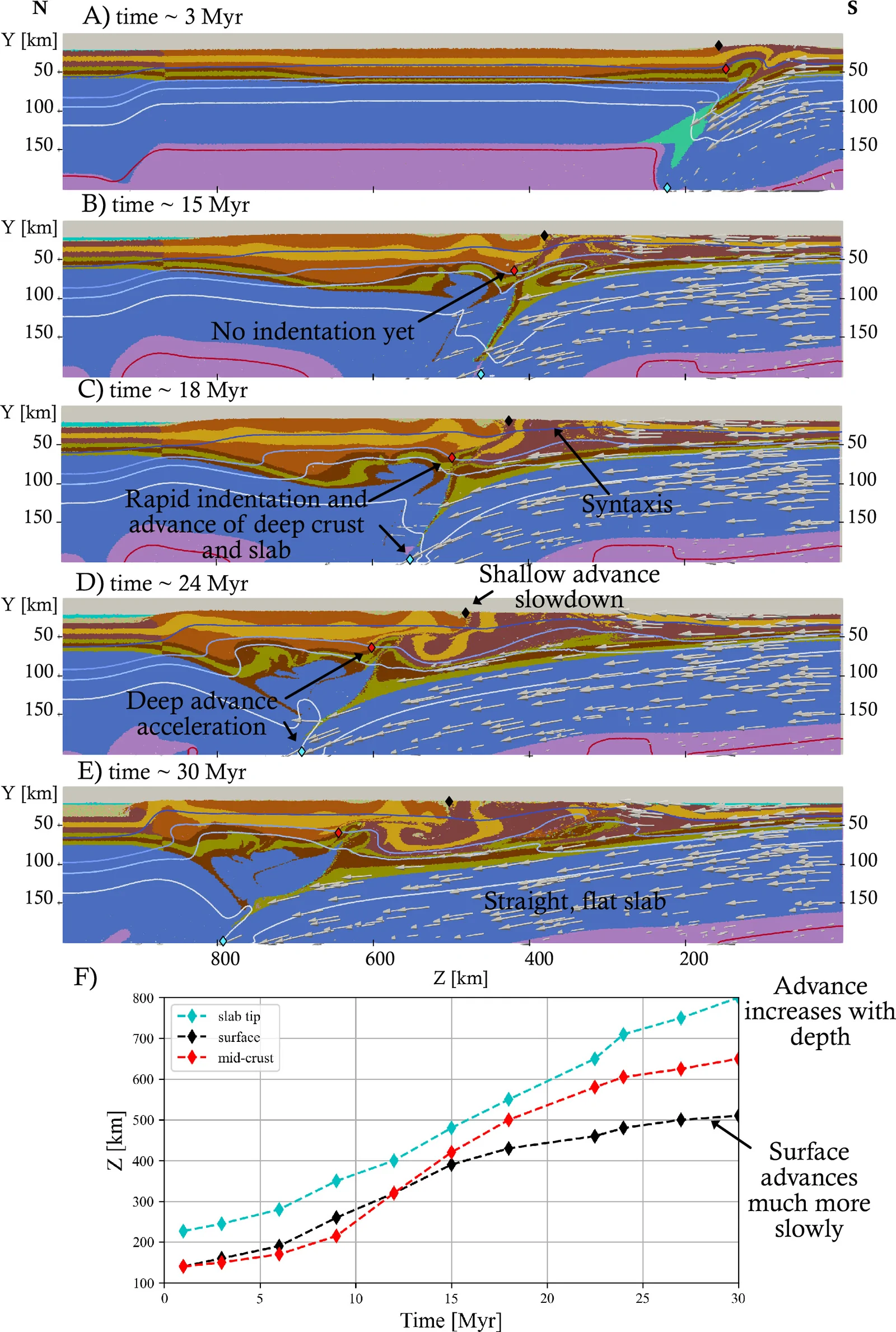
Differential crustal and mantle advance of the Indian plate. **A**-**E**) Evolution of the convergence-parallel position of the India-Eurasia plate boundary at the surface, the upper/lower crust interface, and the indenter slab tip at the X = 400 km slice. North is to the left. Overview in panel **F**. The lower crust and mantle lithosphere of the indenter advance over 250 km further north than the upper crust.

**Extended Data Fig. 4:**
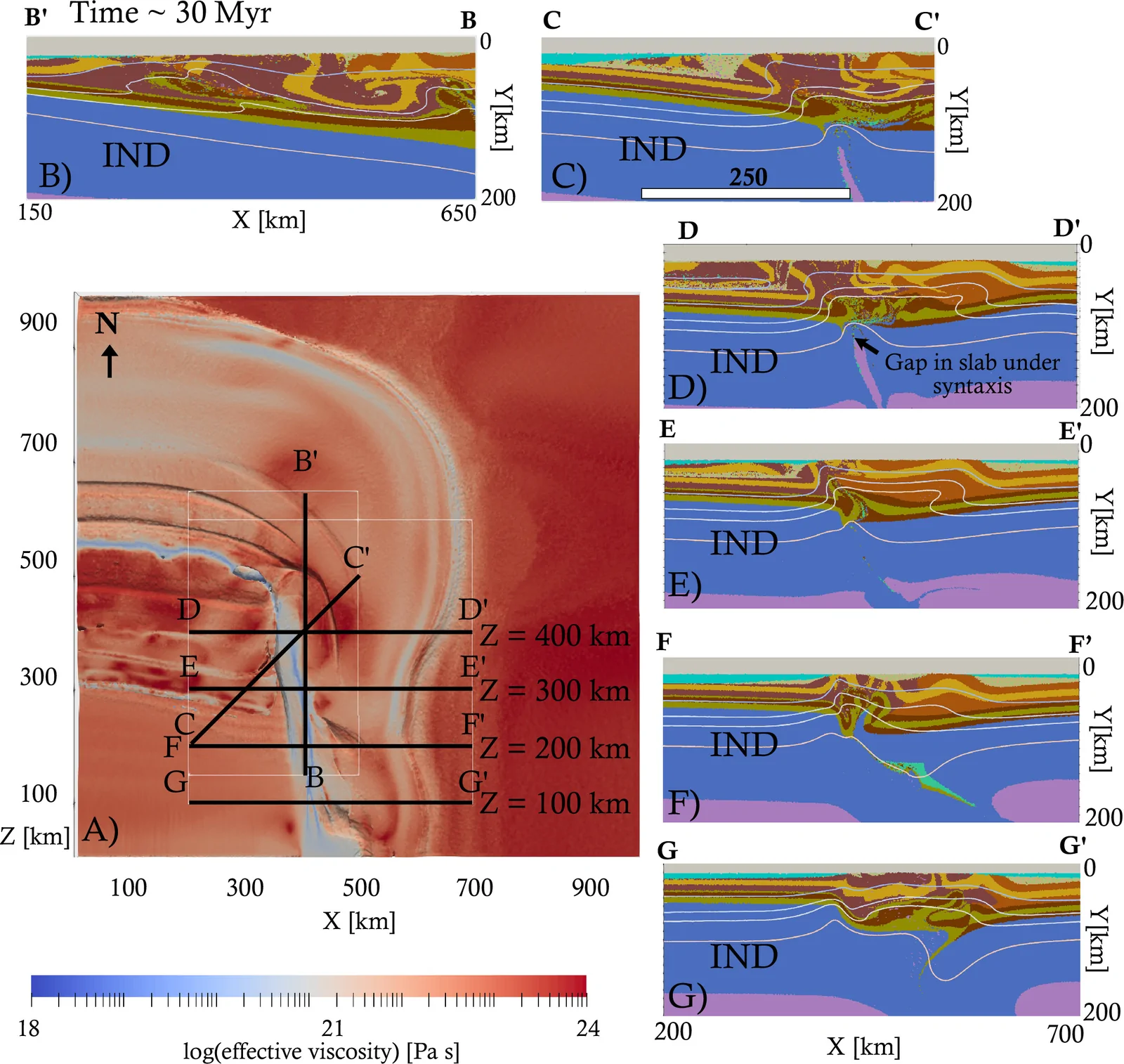
Indian slab geometry under the syntaxis after 30 Myr. **A**) An iso-density contour (2650 kg/m^3^, upper crust) shows the syntaxis in the middle. Sections through two different subsets of the composition field (outlined in white) show the slab geometry under the syntaxis, where the slab is segmented. One NS-cross-section (**B**), one oblique, SW-NE cross-section (**C**) and four E-W cross-sections (**D**-**G**) depicting composition overlain with temperature contours (300, 500, 600 and 800°C).

**Extended Data Fig. 5:**
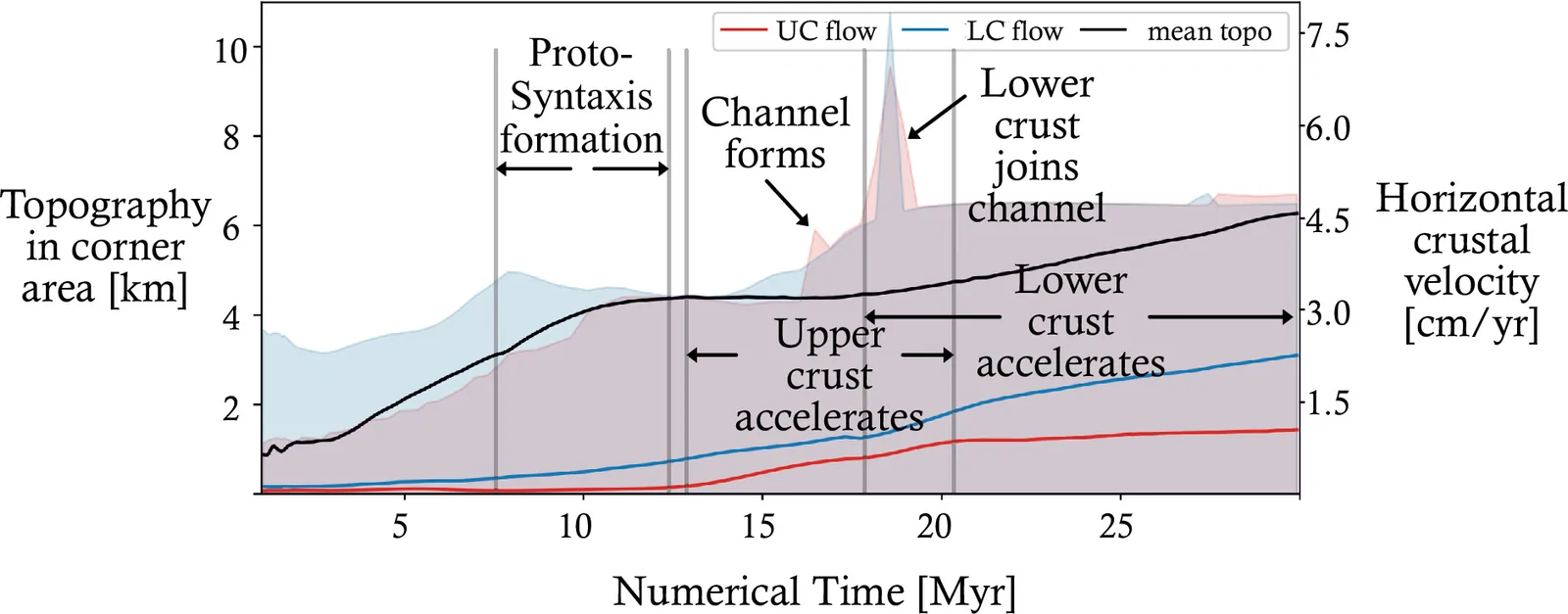
Syntaxis uplift and crustal velocity evolution. The black solid line represents the average topography within the syntaxis area (320 < X < 470 km, 100 < Z < 530 km). The red and blue lines represent the average *horizontal* upper crustal (UC) and lower crustal (LC) flow velocities, respectively. These are averaged through a vertical slice at X = 400 km, between 500 < Z < 1000 km. Maximum flow velocities are shown in a shaded area. A proto-syntaxis forms between 7.5 – 12 Myr. Shortly afterwards, upper crustal flow accelerates, quickly joined by lower crust. The peak at ~18-19 Myr indicates when the channel is most active in the area north of the syntaxis and corresponds to the mid-lower crustal indentation and slab advance pulse as described in the main text.

**Extended Data Fig. 6:**
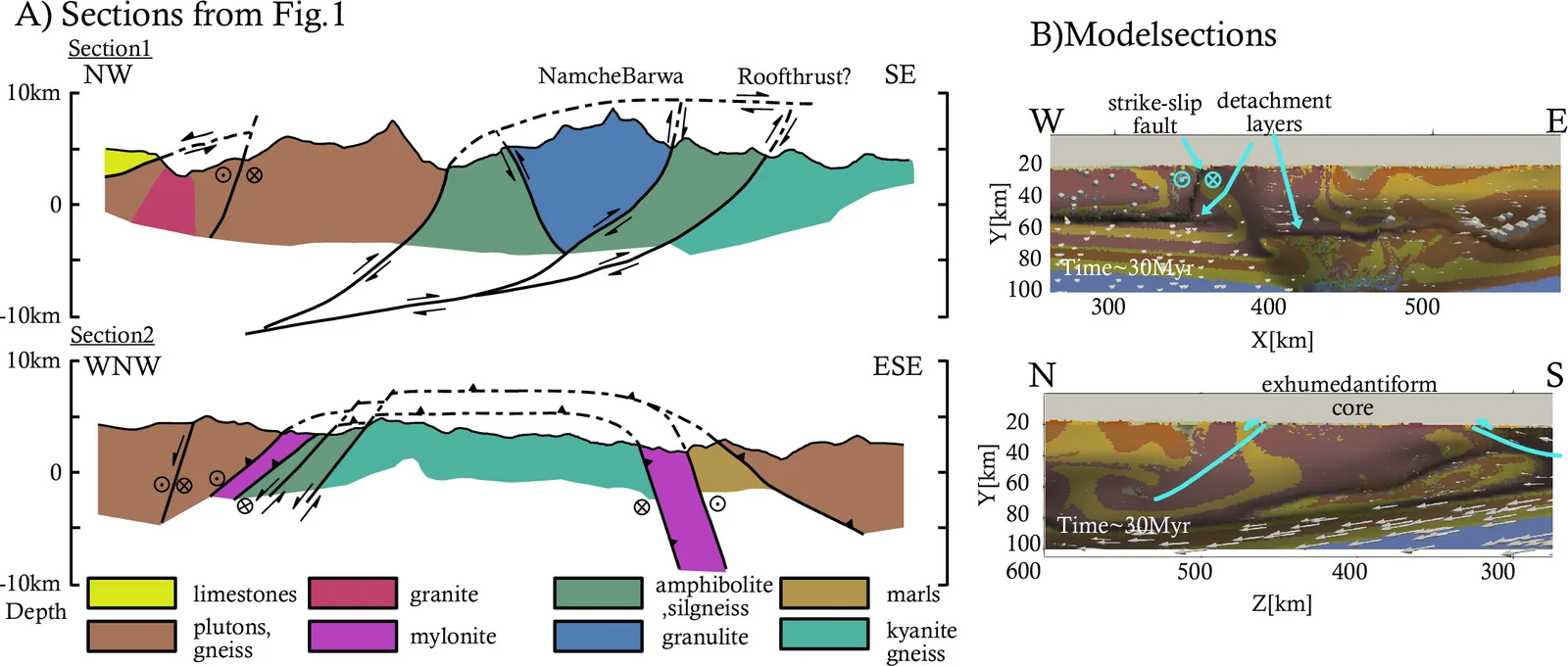
Comparison with Namche Barwa structures. **A**) Simplified geological cross-sections through the EHS at the locations depicted in Fig. 1. Modified from Ding et al.,8. **B**) Zoomed-in rock composition slices with active deformation overlay. The general structure of an exhumed antiformal core is similar, as are the bounding strike-slip faults and oblique thrusts. Note the smaller scale of the sections in panel **A**.

**Extended Data Fig. 7:**
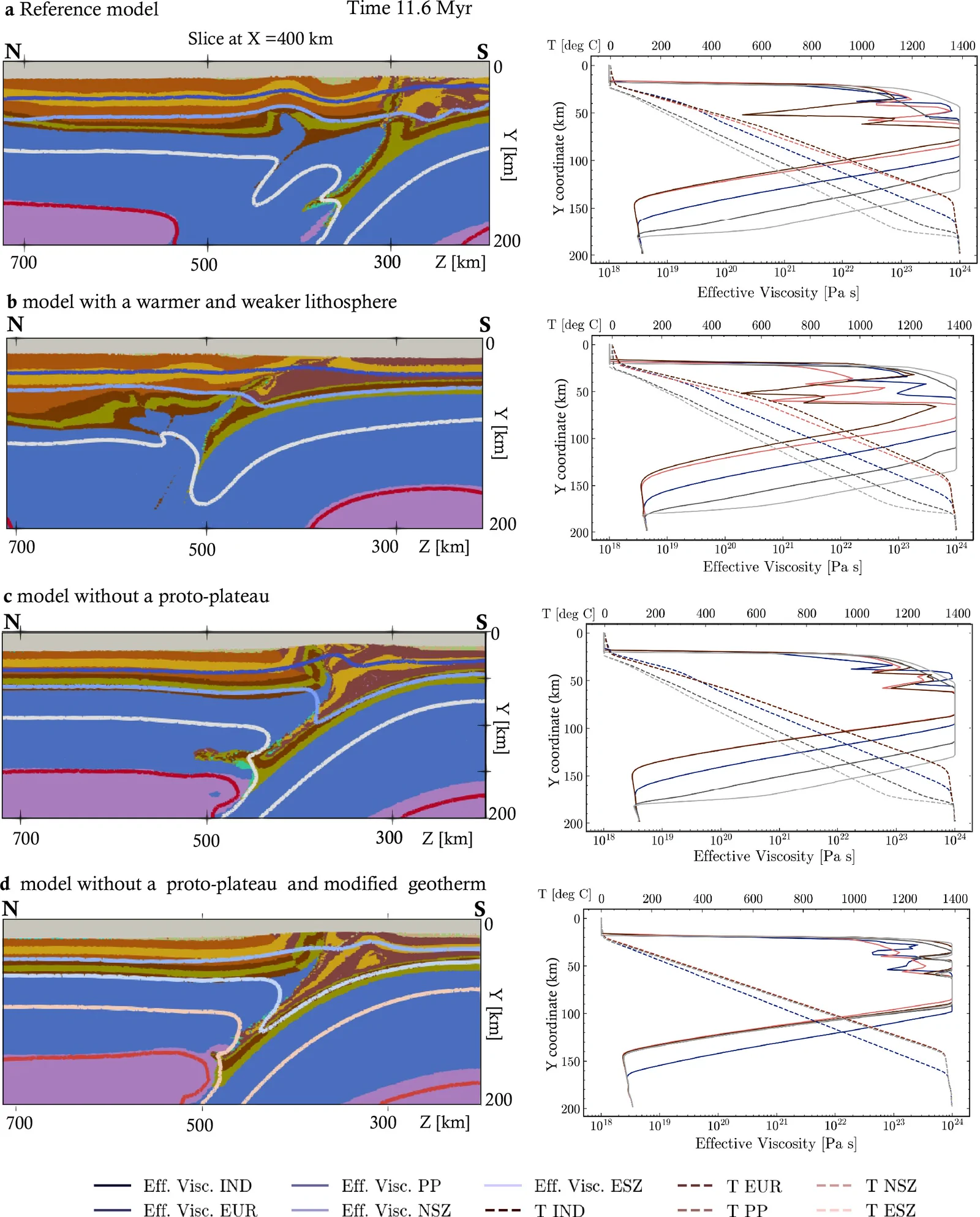
Model comparison with different initial rheological layering. **a-d** models shown by rock composition overlain by temperature contours at 300, 500, 800 and 1300 °C after 11.6 Myr (left) and effective viscosity and temperature profiles (right) at the locations depicted in Extended Data Fig. 1. In the models without a proto-plateau, the overriding plate is too strong preventing slab advance. In the model with a higher thermal gradient the overriding plate is too weak resulting in a too ductile deformation style. IND – India, EUR – Eurasia, PP – proto-plateau, NSZ – northern strong zone, ESZ – eastern strong zone.

**Extended Data Fig. 8:**
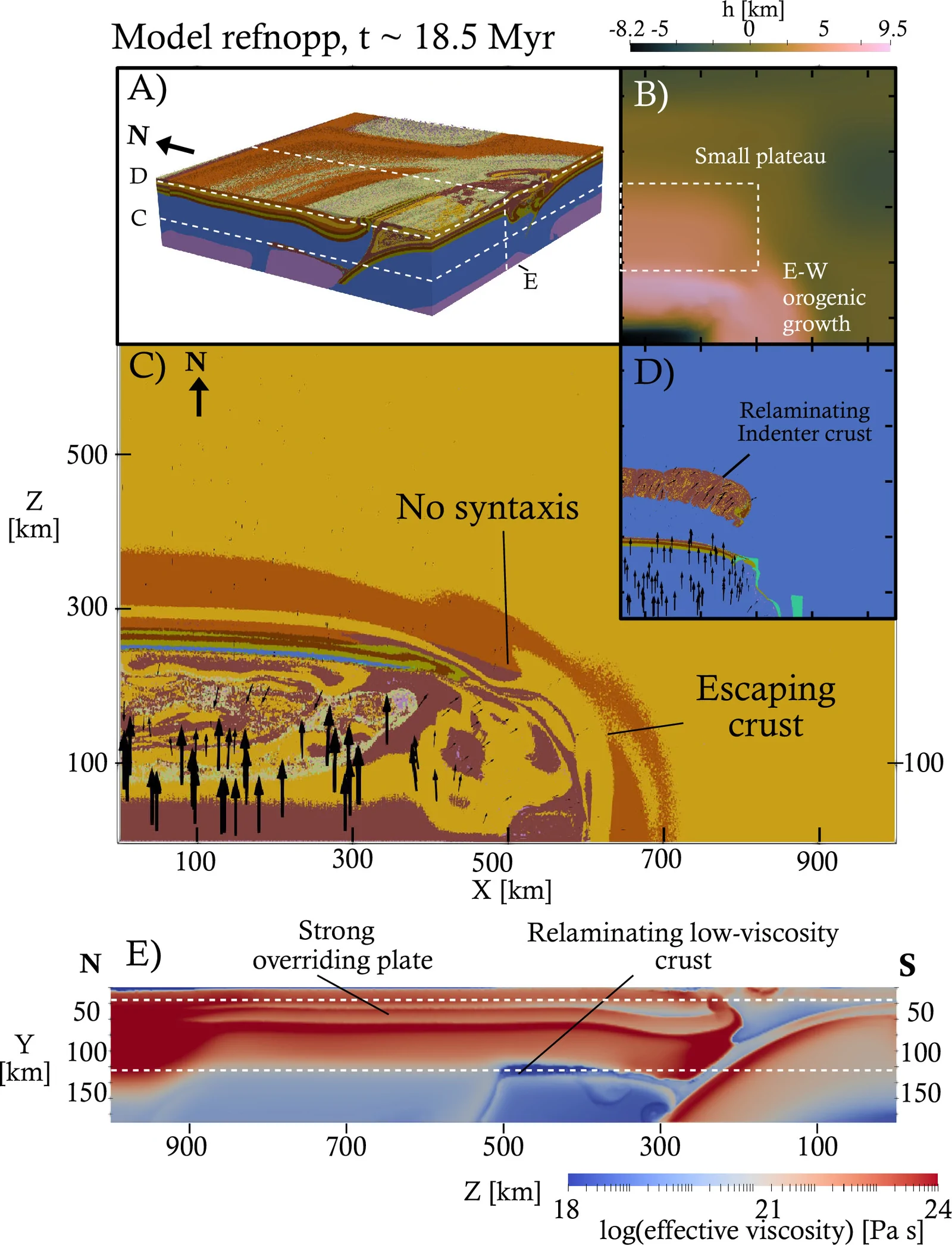
No syntaxis formation when excluding the proto-plateau. Panel **A** shows the oblique top view of rock composition. Panel **B** shows lower topography values in the upper plate compared to the reference model. Locations of horizontal slices at Y = 35 km (panel **C**) and 125 km (panel **D**) and a vertical viscosity slice through X = 400 km (panel E) are shown in white colour. The stronger upper plate hampers northward shortening and indentation, preventing northward plateau growth and crustal channel localization. Parts of the subducted indenter crust relaminates underneath the “Eurasian” plate. Convergence is accommodated by northward subduction and eastward shortening.

Here we show that syntaxis formation, slab dynamics, localized ductile flow at mid–lower crustal depths are tightly interrelated and worked together to establish the current Tibetan–Himalayan orogenic system. This model is based on results of coupled 3D thermomechanical surface process numerical experiments. We did not aim to reproduce specific fault zones of the EHS and southeastern Tibetan Plateau. Rather, our modelling was constrained by first-order observations of the Indian indenter^33–35^ and using a plate convergence rate of 5 cm yr^−1^ that is near the average for the past 30 Myr (refs. ^36,37^). In the reference model, the indenter collides northward with an overriding plate, which has a proto-plateau in its western part. It has been proposed that substantial portion of southern Tibet was raised to 3–4 km elevation before the Indo-Asian collision^38^. Furthermore, two strong zones are defined in the north and east (Extended Data Fig. 1) representing cratonic lithospheres. A sensitivity analysis addressed the effect of the proto-plateau and varied the erosion intensity and thermal structure (compare Supplementary Figs. 1–5 and Extended Data Figs. 2–8).

## Syntaxis formation

**Fig. 2:**
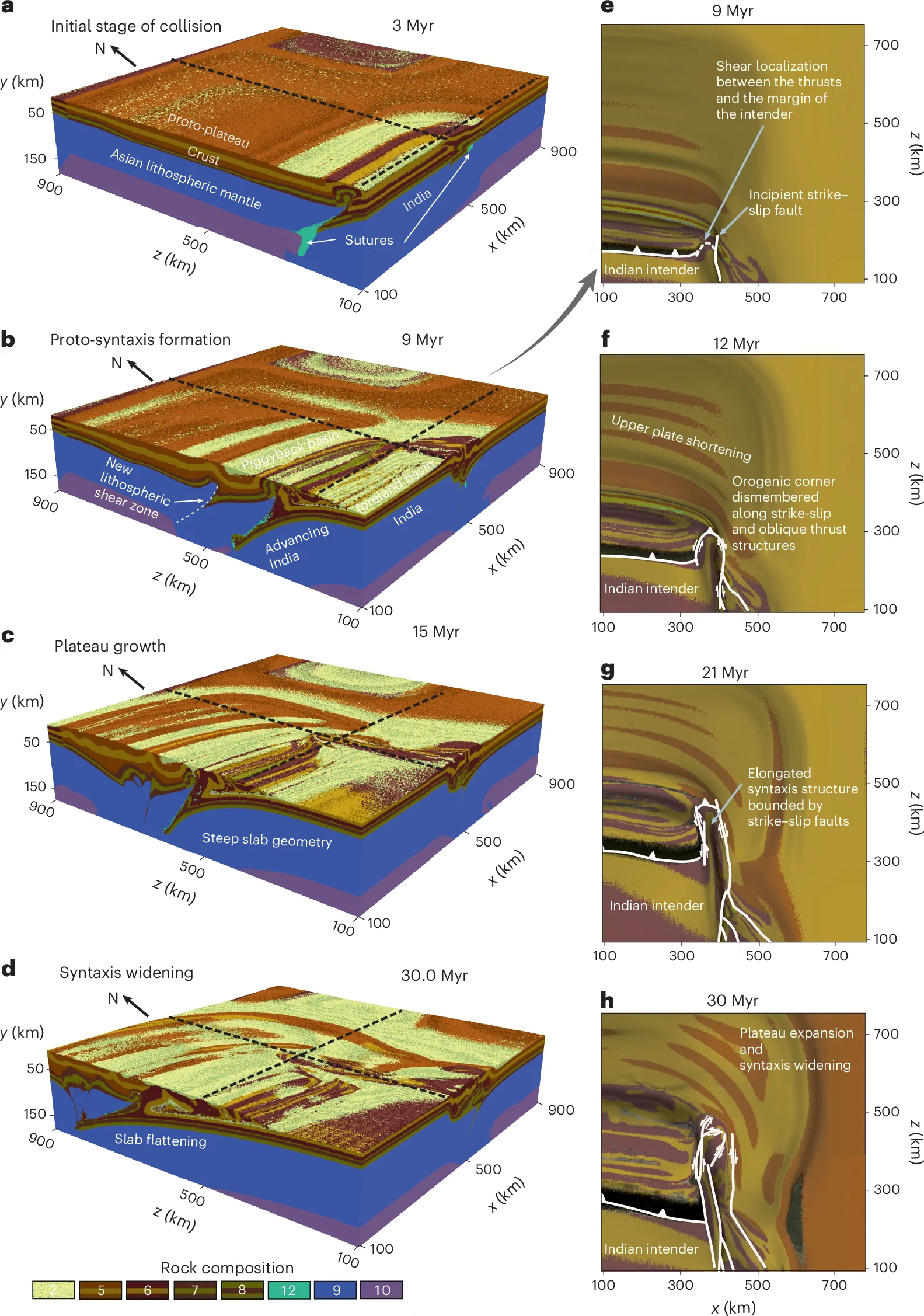
Key stages of model evolution part 1. **a**–**d**, Panels on the left consist of an oblique three-dimensional rock compositional view. The locations of convergence-parallel and moving convergence-perpendicular slices through the corner area (Fig. 3) are indicated with black dashed lines. **e**–**h**, Panels on the right show horizontal slices of rock composition at *y* = 35 km depth with the main structures highlighted in white. Rock composition: (2) sediments, (5) and (6) Eurasian and Indian upper crust, respectively, (7) and (8) Eurasian and Indian lower crust, respectively, (9) lithospheric mantle, (10) asthenosphere, (12) weak suture zone.

**Fig. 3:**
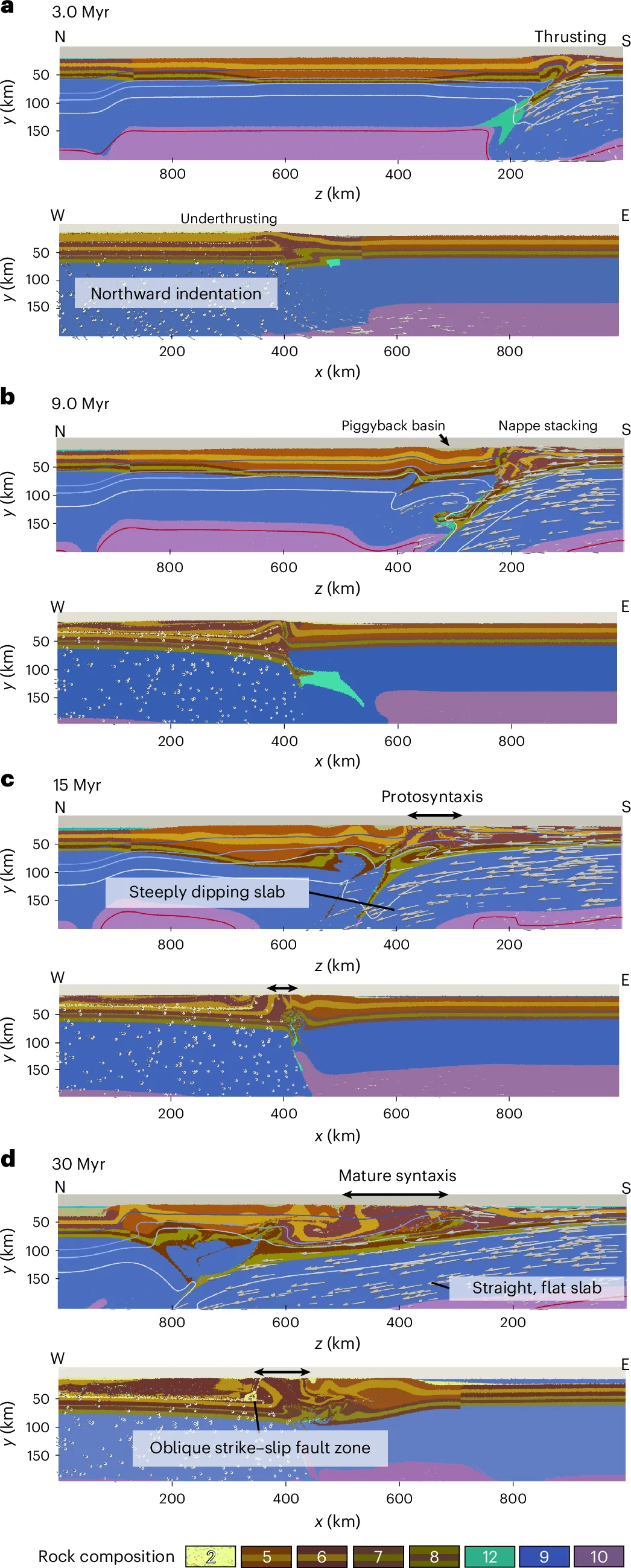
Key stages of model evolution part 2. **a**, Indian underthrusting. **b**, Northward migration of upper plate deformation. **c**, Protosyntaxis formation. **d**, Mature syntaxis formation. The cross-section locations are shown in Fig. 2. The sections are overlain by 3D velocity arrows and temperature contours at *T* = 300, 500, 600, 800 and 1,350 °C. Rock composition: (2) sediments, (5) and (6) Eurasian and Indian upper crust, resp., (7) and (8) Eurasian and Indian lower crust, resp., (9) lithospheric mantle, (10) asthenosphere, (12) weak suture zone.

**Fig. 4:**
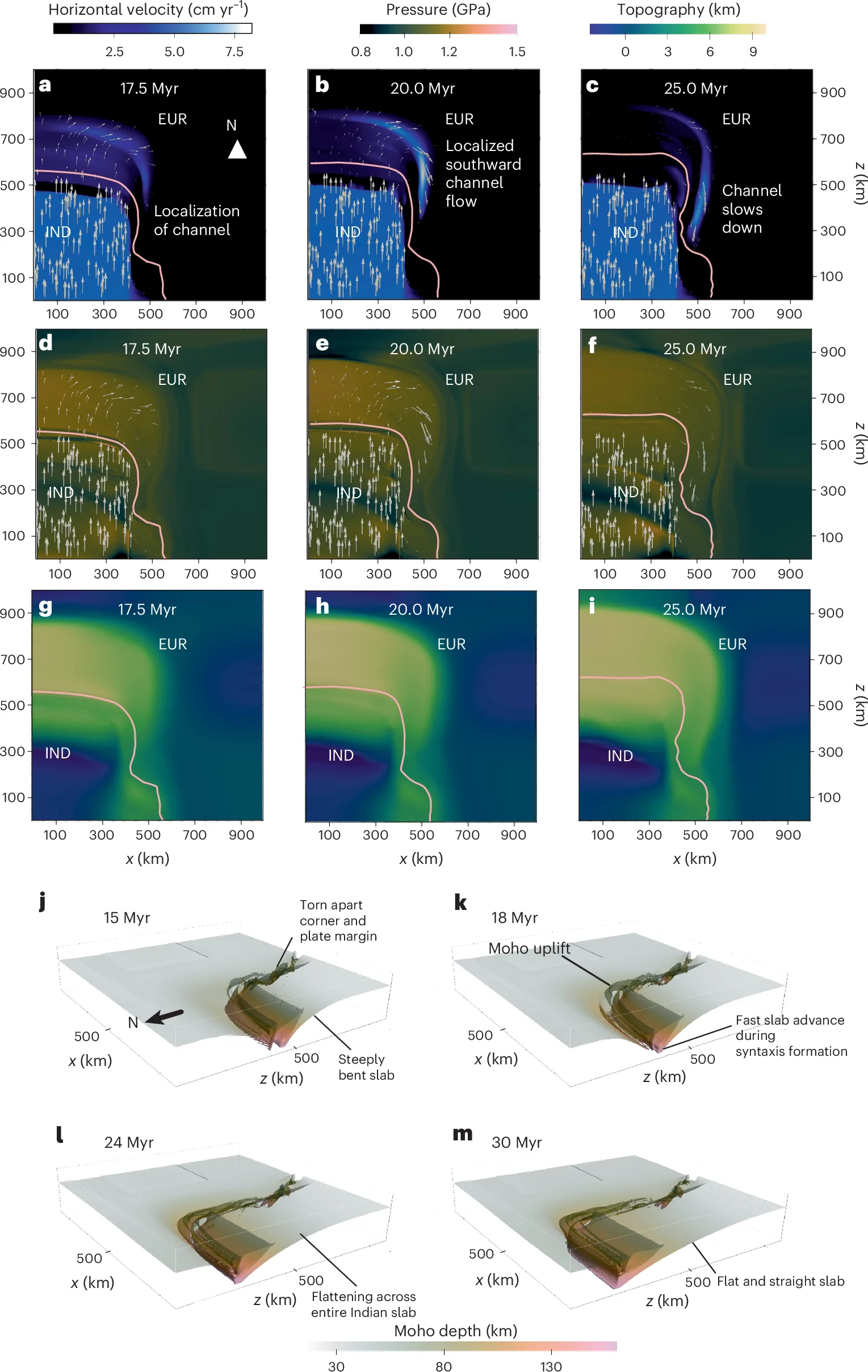
Plateau and syntaxis expansion and Indian slab flattening. **a**–**i**, Horizontal velocity, pressure field at *y* = 60 km and surface topography. The outlines of the Indian and Eurasian plates are indicated in light red. Localized channel forms after 16 Myr and accelerates in response to slab advance and crustal indentation. **j**–**m**, The Moho depth plotted for the reference model. Slab flattening is connected to the fast advance of the Indian mantle lithosphere and lower crust.

During the first 3 Myr of the reference model, subduction localizes along the weak zone between the indenter and Eurasian plate, governing thrusting and upper crustal nappe stacking while the lower crust is coupled to the subducting mantle lithosphere (Figs. 2 and 3 and Supplementary Video 1). Simultaneously, much shallower, eastward underthrusting occurs between the indenter and the Eurasian plate. The initial orogen is relatively high (Fig. 4), symmetric and narrow across strike. The Eurasian crust forms a piggyback basin with a synformal geometry. The orogen becomes slightly asymmetric between 3 and 6 Myr due to faster uplift and erosion along the frontal collision zone. This is visible by tilting of the main antiform and exhumation of the lower crust (green and brown colours in Figs. 2 and 3). Lower crustal exhumation corresponds to an average surface erosion rate of ~ 3.2 mm yr^−1^ between 1.0 and 7.2 Myr numerical time. After 6 Myr numerical time, upper plate deformation migrates northward. A new shear zone localizes with related thrusts and piggyback basins on top by 9 Myr numerical time (Figs. 2 and 3).

Syntaxis formation starts between ~7.5 Myr and 12 Myr, during which the corner of the collision zone is gradually dismembered by the localization of N–S striking, crustal-scale, right-lateral strike–slip shear zones driven by differential plate advance from west to east. The indenter advances further northward in the west due to weaker shear resistance compared to that at the collisional corner and favoured by the weak crust of the proto-plateau (Fig. 2). The advancing indenter leads to further upper plate crustal thickening. A proto-syntaxis forms southeast from the margin of the thickening plateau, consisting of a N- to NNE-plunging antiform (Figs. 2c and 3c). Between 15 and 21 Myr numerical time, the narrow, elongated proto-syntaxis is translated northward by ~250 km with respect to its original location along two strike–slip faults (Fig. 2g). During this elongation, the syntaxis migrates further north than the main collisional front (Fig. 2). After 21 Myr, the syntaxis widens along oblique-slip fault zones until the end of the simulation (Figs. 2 and 3). The syntaxis and plateau widening is further accommodated by thrusts along the eastern plateau rim (Figs. 2 and 3).

### Crustal shear localization and flow patterns

After ~15 Myr model time (that is, during elongation of the proto-syntaxis), the differential plateau growth induces high pressure at the mid- to lower crustal level (*y* = 40–70 km, that is, 20–50 km below the surface) with respect to the less deformed eastern part (Fig. 4). The numerically modelled dynamic pressure difference is ~160 MPa. This pressure gradient allows the weak (that is, 10^18^–10^19 ^Pa s; Extended Data Fig. 1), ductile part of the thickened plateau crust to flow eastward. In the gradually expanding plateau, the weak, ductile crustal mass transport is concentrated in a 30–35 km wide, ~30–40 km deep channel (Fig. 4), reaching a maximum velocity of 7.5 cm yr^−1^ (Fig. 4). The overlying upper crust and underlying lithospheric mantle are decoupled from the ductile crustal deformation. The localized crustal channel continues 400 km eastward between ~16 and 20 Myr, curves around the syntaxis and extends 400 km southward between 20 and 30 Myr. These flow velocities correspond to 4–5 cm yr^−1^ on average. By 25 Myr, the pressure gradient between the northern and southern end of the channel has decreased to ~30 MPa, leading to gradual cessation of mid–lower crustal flow (Fig. 4). The escaping mid–lower crust passes through the area north of the syntaxis in eastward direction between 18–19 Myr numerical time (Fig. 4). By the end of the model run at 30 Myr, the crustal thickness has reached ~70 km in the central part of the orogen and over 100 km where Indian crust is underthrusting (Fig. 3).

### Slab flattening and flow barriers

The slab dip decreases between 15 and 30 Myr numerical time, transitioning into a flat-slab geometry along the entire width of the indenter (Figs. 3 and 4 and Supplementary Video 1). The slab drags the ductile mid–lower crust beneath the syntaxis northward, enabling the Indian mid to lower crust to rapidly indent into the weaker crust of the plateau. Simultaneously, the thick plateau inhibits further northward advance of the Indian upper crust. Between 15 and 30 Myr, the plate boundary in the uppermost crust advances northward with an average rate of 0.87 cm yr^−1^, while the Indian lower crust advances faster northward with the Indian plate tip. Underneath the syntaxis, the slab is segmented and mantle temperature contours bulge up, driven by mantle upwelling (Figs. 3 and 4).

### The role of a proto-plateau

We performed simulations without an initially defined proto-plateau. In these models, neither vigorous crustal flow, nor a syntaxis develop (Extended Data Fig. 8). The collisional front does not advance beyond *z* ~ 200 km, and convergence-parallel deformation stagnates before a plateau has formed. Convergence is instead accommodated by subduction of the Indian mantle lithosphere and convergence-perpendicular buckling of the crust along the eastern margin of the indenter, forming a progressively wider, north–south striking orogen. At ~15 Myr, some crustal material decouples from the downgoing plate and relaminates to the base of the Eurasian lithosphere.

### Conditions for syntaxis formation

**Fig. 5:**
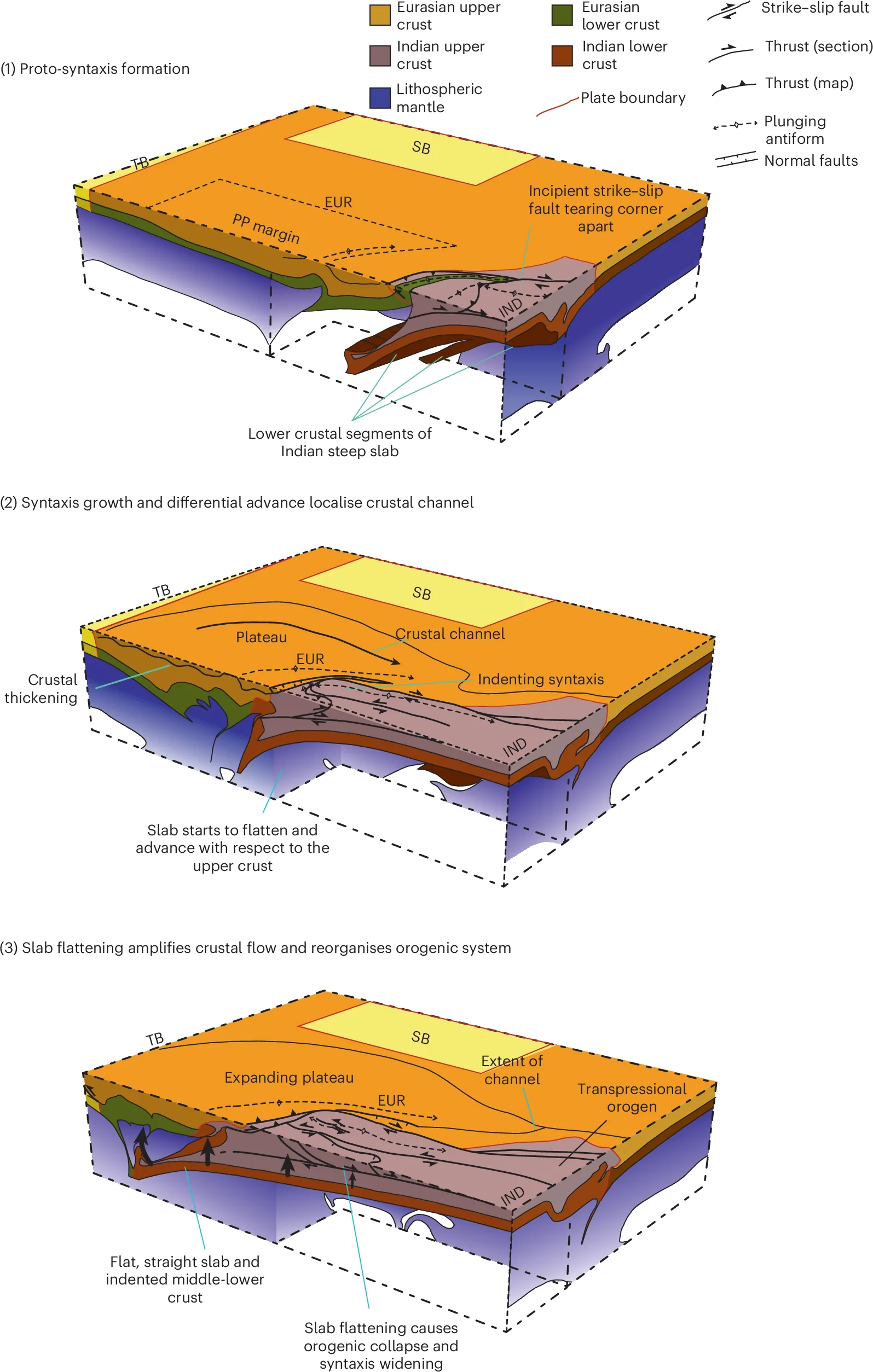
Conceptual model for syntaxis formation and evolution. Stage 1: development of a proto-syntaxis and high, narrow topography in front of collision zone. Stage 2: thickened crust of the plateau flows east and southward in a localized channel in response to differential advance (both vertically and along strike), while the syntaxis indents northward and becomes elongated. Stage 3: slab flattening governs the subsequent evolution, causing orogenic collapse and widening of the syntaxis, which results in a more complex and diverse faulting pattern. IND: Indian indenter, EUR: Eurasian Plate, TB: Tarim Basin, SB: Sichuan Basin, PP: proto-plateau.

The model series show that a pre-defined proto-plateau^38^ in the upper plate is required for the formation of an orogenic syntaxis during indenter collision controlling a weak and decoupled ductile crustal layer between the brittle upper crust and the mantle lithosphere. This layer can quickly mobilize in response to the advancing indenter (Fig. 5). The corner area then tears along localized, N–S striking, strike–slip fault zones, while more ductile buckling occurs in the plateau area to the north. The strike–slip fault zones allow the syntaxis to indent into the plateau area, amplifying the observed curvature of the orogenic front.

The style of deformation in the corner depends on the crustal rheological contrast between the indenter and Eurasian plate. A medium strength contrast (*T*_Moho_ contrast of 100°C) between the indenter and the Eurasian plate promotes syntaxis formation (Fig. 2 and Extended Data Fig. 1). Models with lower (*T*_Moho_ contrast ~60 °C) strength contrast lead to steep subduction and negligible plateau growth (Extended Data Fig. 7). Models with a hotter overriding plate (*T*_Moho_ contrast 200 °C) show excessive advance, insufficient subduction and, crucially, no decoupling between the mid-crust interface in the indenter.

The proto-syntaxis in our model forms between the nappe stack to the west and the eastern margin of the indenter and connected strike–slip fault zones in the east (Figs. 2–5). The transpressive zone that is formed along the nappe stack and its associated shear zones is critical to proto-syntaxis development. By accommodating north–south folding and enabling indentation into the overriding plate, it drives the structural evolution of the EHS (Figs. 2–5). In models where strike–slip faults are not well developed (Extended Data Figs. 7 and 8), the syntaxis instead passively follows the plateau expansion.

### Slab flattening under Tibet

The evolution of the syntaxis in our models is tightly linked to slab flattening starting at ~17 Myr (Figs. 3 and 4). These processes are initiated by differential, northward advance of the indenter both along strike and with depth (Figs. 2–4 and Extended Data Fig. 3). This decoupled advance and indentation triggers crustal strain localization around the corner of the indenter (Figs. 2–5 and Extended Data Figs. 3 and 5). To accommodate the rapid northward advance of the slab, the plateau lithosphere bends and retreats to the north (Figs. 2–5, Extended Data Fig. 3). The eastern strong zone prohibits advance to the east and promotes E–W buckling and ductile crustal deformation around the syntaxis towards the south (Extended Data Fig. 2).

Previous studies using a simplified homogeneous overriding plate crust have explained the flat geometry of the Indian slab through buoyant rise of the continental part of the slab after the oceanic portion has detached^39,40^. Other models for the Tibetan plateau evolution emphasized the role of overriding plate mantle delamination to explain magmatism and the uplift history of Tibet^41,42^. Our models show that slab flattening can occur without underplating through the interaction with Tarim lithosphere.

Recent seismological and tomographic studies have reported that the Indian slab is clearly visible beneath the western and central Himalayas and Burma terrane and it is probably segmented under the syntaxis^33,34,43^. For comparison, we have selected similar slices through the syntaxis area in our reference model (Extended Data Fig. 4). The observed east and northward subduction geometry transition from relatively steep to flat subduction is similar to observational data (Fig. 5 in ref. ^34^). The proposed indentation of rigid India under the syntaxis is also obtained in our reference model, where isotherms bulge upward at the plate boundary zone (Fig. 3), contributing to syntaxis uplift^43^.

### Comparison with Eastern Himalayan syntaxis and Tibet

The final syntaxis structure in our reference model has some striking similarities with the EHS (Fig. 1 and Extended Data Fig. 6). The modelled syntaxis starts as a transpressional shear zone connected with the collisional front to the west and eastern oblique subduction zone to the southeast. The Indian upper crust in this initial structure is subsequently folded before indenting the Eurasian plate (Figs. 2 and 3). Our models therefore favour the second proposed theoretical model of Ding et al.^8^, where a segment of folded Indian crust indents the Asian margin as the syntaxis develops (compare Fig. 2 with Fig. 7b in Ding et al.^8^).

Given the low strength of the Tibetan Plateau^23^ and fast advance of India^37^, we argue that the EHS moved northward at a rate comparable to the advance of India as suggested by King et al.^17^ and supported by various geomorphic indicators^44^. Northward migration of the EHS is also reflected in the diachronous cooling history of the Mogok metamorphic belt in Myanmar, directly south of the syntaxis^45^. This evidence for a northward younging trend of geomorphic features challenges that the syntaxis has stayed stationary with respect to India and Indochina^12^. Our models corroborate the inference that relative transversal flow of Eurasian crust over the subducting Indian lithosphere and continuous subduction increase syntaxis curvature^12^. Our models also show similar clockwise rotating upper crustal velocities (Supplementary Video 1) as observed in Tibet^46,47^. The indentation by the syntaxis enhances extrusion. This extrusion involves strain localization of crustal-scale strike–slip shear zones and ductile crustal flow in narrow channels bounded by shear zones (Fig. 4), similar to the Ailo Shan zone in SE Asia^48^. Furthermore, the listric thrust faults in the eastern plateau rim in the reference model (Extended Data Fig. 2) resemble the large thrusts in southeastern Tibet, such as the Muli thrust^32^.

The crustal channel beneath the Tibetan Plateau has been controversial^26–38^. Our simulated ductile crustal flow differs from the previously proposed channel flow models^26–30^. In our models the ductile lower crust allows the decoupling between crust and mantle and the localization of the deformation in the crust (Figs. 2 and 3). The pressure difference allows the weak, ductile part of the thickened plateau crust to flow around the syntaxis, but it is limited by a 30–35 km wide, ~30–40 km deep zone creating a localized pressure-driven flow (Fig. 4b). In agreement with Pitard et al.^32^, the large shear zones developed in the model, similar to the Muli thrust, limit the flow toward the east. Our 3D models show that only transient, localized channels form bounded by strike–slip and thrust faults (Figs. 3 and 4).

In our models, the maximum flow velocity in the centre of the spontaneously opening crustal channel is ~ 7.5 cm yr^−1^ (Fig. 4b). The model travel distance of mid–lower crustal material is up to 800 km. An extensive analysis of thermochronological data in the southeastern Tibetan Plateau revealed that transient crustal flow probably controlled exhumation of metamorphic complexes between 30 and 20 million years ago along the Red River and Sagaing fault systems^49^. The channel in our simulations is also active over an ~ 10-Myr period (Fig. 4), but we did not observe the northward counter flow of lower crust as suggested^49^. This is probably due to the absence of pre-existing crustal weak zones, partial melting or suture zones in that area of our models.

## Methods

### Modelling of continental corner collision

#### Modelling approach

The geodynamic, fully coupled, three-dimensional, thermomechanical surface process C code I3VIS-FDSPM combines a finite-difference method with a marker-in-cell technique on a staggered Eulerian grid^50–52^. The code solves the conservation of momentum, mass and energy equations on the Eulerian grid, while rock units—markers—are advected through the fixed Eulerian grid following the velocity field interpolated from the surrounding fixed grid points. The rocks deform following non-Newtonian visco-plastic rheologies (Extended Data Table 1). The code takes into account frictional, adiabatic and radiogenic heat production. The complete description is provided in previous works^50,51^.

#### Numerical model design

The models consist of 501 × 501 × 101 (*x*, *z*, *y*) Eulerian nodes with a uniform 2-km grid step in each direction, corresponding to a model domain size of 1,000 × 1,000 × 200 km^3^. Each cell contains eight markers. The model domain is resolved using 25 million Eulerian nodes through which 200 million markers advect the rock properties. The reference model consists of four continental plates: the indenter, representing part of India, is located in the southwest corner and transitions into the slightly weaker Eurasian plate to the north and east (Extended Data Fig. 1a). To mimic the effects of the cratonic Tarim and Sichuan basins, we use a northern and eastern strong zone (NSZ and ESZ). Lithospheric structure thicknesses of the Indian indenter plate consist of 20 wet quartzite upper crust, 15 km wet plagioclase lower crust and 105 km of lithospheric mantle (Extended Data Fig. 1 and Extended Data Table 1). For Eurasia, these thicknesses are 23, 17 and 80 km, respectively. The NSZ and ESZ have the same crustal thicknesses as the indenter, but thicker lithospheric mantles of 125 and 145 km, respectively. This makes the ESZ and NSZ colder and therefore stronger than the other plates (Extended Data Fig. 1b). To initiate subduction and ensure that India is the lower plate in each direction, two weak zones composed of serpentinized mantle are emplaced between the indenter and Eurasia to the north and east. These serpentinized mantle weak zones extend from the base of the crust to the base of the lithospheric mantle at an angle of 30° (Extended Data Fig. 1a) and represent the Neotethys suture and Burmese subduction interface. In front of the indenter and within the Eurasian domain, a proto-plateau is defined between *x* = 0–400 km and *z* = 340–740 km, which has a 35-km-thick upper crust, 10-km-thick lower crust and 75-km-thick lithospheric mantle. The effect of this proto-plateau is discussed in the main text. Finally, we use a 20-km-thick layer of sticky air to allow topography to build (Extended Data Table 1).

The indenter initially has a three-segment geotherm (300 °C at the mid-crust interface, 400 °C at the Moho), whereas Eurasia (500 °C at the Moho) and the proto-plateau (600 °C at the Moho) have a two-segment geotherm. The strong zones have an initial linear geotherm from 0 °C at the surface to 1,350 °C at the lithosphere–asthenosphere boundary (LAB), which is the same temperature for all plates. An adiabatic gradient of 0.5 °C km^−1^ is applied in the asthenospheric mantle. The model sides are insulating, that is, zero horizontal heat flux. An infinity-like external temperature is prescribed at the lower boundary, implying a temperature of 1,216 °C at *y* = 400 km. Therefore, the temperature and vertical heat flux at the resolved lower boundary can vary. Similarly, we impose an external free slip boundary condition at *y* = 400 km allowing for subduction through the lower boundary despite our limited vertical model extent. The side and back boundaries are free slip. We apply a push velocity of 5 cm/yr to the Indian indenter, which tapers to 0 cm yr^−1^ between *x* = 400–500 km (Extended Data Fig. 1a). The material influx is compensated with an outflux of air at the top of the model domain of 0.041 cm yr^−1^ and an outflow of mantle through the lower boundary of 0.85 cm yr^−1^ to conserve volume.

#### Visco-plastic rheological model

We use an effective viscosity formulation to simulate the plastic (‘brittle’) and viscous (ductile) strength of the modelled rocks, using the properties in Extended Data Table 1^53–55^. The viscous flow laws for dislocation and diffusion creep are harmonically averaged into a ductile viscosity ${{{\eta }}}_{\mathrm{ductile}}$

$$\frac{1}{{{{\eta }}}_{{\rm{ductile}}}}=\frac{1}{{{{\eta }}}_{{\rm{newt}}}}+\frac{1}{{{{\eta }}}_{{\rm{powl}}}}$$

Here ${{{\eta }}}_{\mathrm{newt}}$ and ${{{\eta }}}_{\mathrm{powl}}$ represent the contributions from diffusion and dislocation creep. For the crust and sediments, these are calculated as

$${{{\eta }}}_{{\rm{newt}}}+\frac{{A}_{d}}{{2{\rm{\sigma }}}_{{\rm{cr}}}^{n-1}}\exp \left(\frac{{E}_{a}+{{PV}}_{a}}{{RT}}\right)$$

$${{{\eta }}}_{{\rm{powl}}}+\frac{{A}_{{\rm{d}}}^{1/n}}{2}\exp \left(\frac{{E}_{a}+{{PV}}_{a}}{{nRT}}\right){{{\dot{\varepsilon }}}}_{{\rm{II}}}^{\frac{1}{n-1}}$$

Where ${A}_{\mathrm{d}}$ is the prefactor for both creep laws, ${{\rm{\sigma }}}_{\mathrm{cr}}$ defines the transition stress between diffusion and dislocation creep, ${E}_{\mathrm{a}}$ and ${V}_{\mathrm{a}}$ represent the activation energy and volume, n is the stress exponent and *R* and *T* are the universal gas constant and absolute temperature. ${{{\dot{\varepsilon }}}}_{\mathrm{II}}$ is the second invariant of the strain rate tensor. ${A}_{\mathrm{d}},\,{E}_{\mathrm{a}},{V}_{\mathrm{a}}$ and n are determined from experiments.

For mantle rocks, a different diffusion creep flow law is used, now also considering grain size evolution

$${{{\eta }}}_{{\rm{newt}},{\rm{mantle}}}=\frac{{A}_{{\rm{d}},{\rm{GSE}}}}{2}\exp \left(\frac{{E}_{a}+{{PV}}_{a}}{{RT}}\right){\left(\frac{{\rm{\pi }}{\rm{r}}}{2}\right)}^{m}$$

Here ${A}_{\mathrm{d},\mathrm{GSE}}$ now depends on the deformation regime, r is the grain interface curvature and m the grain size exponent. The curvature is coupled to the actual grain size through the factor $\left({\rm{\pi }}/2\right)$^56^.

After calculating the ductile rheology, it is merged with the brittle part to obtain an effective visco-plastic rheology as follows:

$${{{\eta }}}_{{\rm{ductile}}}\le \frac{C+{\rm{\mu }}\left({{\varepsilon }}\right)P}{2{{{\dot{\varepsilon }}}}_{{\rm{II}}}}$$

Here C is the tensile strength of rocks at zero pressure, *P* is pressure, ${{{\dot{\varepsilon }}}}_{\mathrm{II}}=\sqrt{{({\dot{\varepsilon}}}_{\mathrm{ij}}{\dot{\varepsilon }}_{\mathrm{ij}})/2}$ is the second invariant of the strain rate tensor and ${\rm{\mu }}$ is the friction angle that decreases over a pre-defined interval of accumulated plastic strain ${\varepsilon }_{{\rm{pl}}}$ from the static friction coefficient ${\mu }_{0}$ to the weakened value ${\mu }_{1}$ (Extended Data Table 1).

$$\mu =\left\{\begin{array}{c}{\mu }_{0},\\ {\mu }_{0}\bullet \frac{{\varepsilon }_{{\rm{pl}}}-{\varepsilon }_{0}}{{\varepsilon }_{1}-{\varepsilon }_{0}}\\ {\mu }_{1}\end{array}\right.\begin{array}{c}{\rm{for}}{\;\varepsilon }_{{\rm{pl}}}\le {\varepsilon }_{0}\\ {\;\;\;\;\;\;\,\rm{for}}{\;\varepsilon }_{0} < {\varepsilon }_{{\rm{pl}}} < {\varepsilon }_{1}\\ {\rm{for}}{\;\varepsilon }_{{\rm{pl}}}\ge {\varepsilon }_{1}\end{array}$$

#### Surface process model

For the surface processes in our model, we use the Finite Difference Surface Process Model, which is fully coupled to the thermomechanical part of the code as described in earlier works^52^. This code takes into account hillslope diffusion, erosion, sedimentation and advective transport of the topographic surface h. Sediments formed through erosion are transported into forming basins in a mass-conservative manner, which most other surface process models do not. This is critical for regional geodynamic studies. The surface evolves following the governing equation

$$\frac{\partial h}{\partial t}+{u}_{H}\cdot {\nabla }_{H}{\rm{h}}={u}_{V}+{\nabla }_{H}\cdot \left({{\kappa }}\left({\bf{x}}\right)\right){\nabla }_{H}{\rm{h}}$$

Where ${u}_{H}$ is the horizontal velocity field (${u}_{x},\,{u}_{z})$, ${{\kappa }}\left({\bf{x}}\right)$ denotes the diffusivity depending on position ***x*** (*x*, *y*, *z*). However, here we assume a constant diffusivity ${{{\kappa }}}_{c}$ across the surface domain, yielding

$$\frac{\partial h}{\partial t}+{u}_{H}\cdot {\nabla }_{H}h={u}_{V}+{{{\kappa }}}_{c}{\nabla }_{H}^{2}h$$

Where the topographic curvature is denoted with ${\nabla }_{H}^{2}h$. Erosion takes place when the curvature is negative, while sedimentation occurs when it is positive. The governing equation is solved using a first-order operator-splitting approach, which involves calculating the advective and diffusive terms sequentially.

It should be noted that our surface process model does not account for fluvial processes (that is, stream power law). The Tibetan–Himalayan system is drained by many major rivers, especially in the corner (Fig. 1). We expect that fluvial erosion strengthens the tectonics–surface-process coupling that is important for syntaxis formation.

## Online content

Any methods, additional references, Nature Portfolio reporting summaries, source data, extended data, supplementary information, acknowledgements, peer review information; details of author contributions and competing interests; and statements of data and code availability are available at https://doi.org/10.1038/s41561-026-02045-7.

## Supplementary information

Supplementary Information Supplementary Figs. 1–5 and discussion.

Supplementary Video

## Data Availability

All the relevant data and model outputs presented in this study are available via Zenodo at https://doi.org/10.5281/zenodo.20289646 (ref. ^57^) and in the Supplementary Information.

## Extended data

**Extended Data Table 1:** Rheological parameters for the models

	Sediments	Upper cont. Crust	Lower cont. Crust	Lithospheric mantle	Asthenospheric mantle	Mantle weak zone
Flow law	Wet qtz.	Wet qtz.	Plag.	Dry olivine	Dry olivine	Wet olivine
Density ${\rho }_{0}$ [kg m^−3^]	2600	2700	2800	3300	3300	3300
Pre-exponential factor, $\frac{1}{{A}_{D}}$ [Pa^n^ s^−1^]	$1.97\cdot {10}^{17}$	$1.97\cdot {10}^{17}$	$4.8\cdot {10}^{22}$	$1.10\cdot {10}^{16}$	$1.10\cdot {10}^{16}$	$1.10\cdot {10}^{16}$
Activation energy, E [kJ mol^−1^]	154	154	238	530	530	530
Activation volume, V [J bar^−1^]	0	0	0	2.6	2.6	2.6
Stress exponent *n*	2.3	2.3	3.2	3.5	3.5	3.5
Cohesion [MPa]	1	1	1	3	3	3
Friction coefficient range $\left[{\mu }_{0},{\mu }_{1}\right]$	[0,0]	[0.2, 0.1]	[0.2, 0.1]	[0.6, 0.0]	[0.6, 0.0]	[0, 0]
Strain weakening interval $\left[{\varepsilon }_{0},{\varepsilon }_{1}\right]$	[0.5, 1.5]	[0.25, 1.0]	[0.25, 1.0]	[0, 0.5]	[0, 0.5]	[0, 0.5]
Radioactive heat production *H*_*r*_ [μW m^−3^]	2	1	0.5	0.022	0.024	0.026
Thermal expansivity α [°K^−1^]	$3\cdot {10}^{-5}$ ${10}^{-5}$	$3\cdot {10}^{-5}$	$3\cdot {10}^{-5}$	$2\cdot {10}^{-5}$	$2\cdot {10}^{-5}$	$2\cdot {10}^{-5}$
Compressibility coefficient [kbar^−1^]	$1\cdot {10}^{-3}$	$1\cdot {10}^{-3}$	$1\cdot {10}^{-3}$	$6\cdot {10}^{-4}$	$6\cdot {10}^{-4}$	$6\cdot {10}^{-4}$
Thermal conductivity *k* [W m^−1^K^−1^]	$\left(0.64+\frac{807}{T+77}\right)$ $\exp \left(4\cdot {10}^{-6}P\right)$	$\left(0.64+\frac{807}{T+77}\right)$ $\exp \left(4\cdot {10}^{-6}P\right)$	$\left(1.18+\frac{474}{T+77}\right)$ $\exp \left(4\cdot {10}^{-6}P\right)$	$\left(0.73+\frac{1293}{T+77}\right)$ $\exp \left(4\cdot {10}^{-6}P\right)$	$\left(0.73+\frac{1293}{T+77}\right)$ $\exp \left(4\cdot {10}^{-6}P\right)$	$\left(0.73+\frac{1293}{T+77}\right)$ $\exp \left(4\cdot {10}^{-6}P\right)$

Rheological parameters for the reference model^53–55^. All units displayed here have a specific heat capacity of 1000 J kg^−1^ K^−1^. Sticky air has a density of 1 kg m^−3^ and a constant viscosity of 10^−18 ^Pa s.

## Code availability

The numerical code I3ELVIS is available via Zenodo at https://doi.org/10.5281/zenodo.20286619 (ref. ^58^). 3D data visualization was carried out by Paraview.
