# Supplementary material for: Eastern Himalayan syntaxis formation controlled by slab flattening and crustal shear localization
Source: Nat Geosci. 2026 Jul 20;19(9):1123–31. doi: 10.1038/s41561-026-02045-7 (PMC13541619; doi:10.1038/s41561-026-02045-7)
Supplement: Supplementary file 1 [file 41561_2026_2045_MOESM1_ESM.pdf]

nature geoscience

Supplementary information

https://doi.org/10.1038/s41561-026-02045-7

Eastern Himalayan syntaxis formation controlled by slab flattening and crustal shear localization

In the format provided by the authors and unedited

Supplementary Information for the manuscript titled "Eastern Himalayan syntaxis formation controlled by slab flattening and crustal shear localization" by van Agtmaal, Balazs, Willett, May and Gerya.

Supplementary Video:

Supplementary Video 1: https://youtu.be/KJVqdtfFxzM

Description:

Top panel: North is to the left. Two slices at x = 100 and x = 500 km showing rock composition. Crust and sediments are semi-transparent; mantle is made transparent. A contour of the crust-mantle interface (Moho) is shown, with its depth projected on it. The velocity of the upper crust is shown to highlight its escape flow towards the east. Bottom panels: Top view showing the model surface, plotting the topography and uplift rates. This topography is not corrected for the background subsidence described in the Methods section.

Main model stages are annotated: Stage A: Initial deformation, mountain building Stage B: Proto-syntaxis formation in the corner Stage C: Plateau growth, fast ductile crustal channel flow around the corner, slab flattening Stage D: Syntaxis widening, slower crustal flow, and plateau collapse.

Additional figures

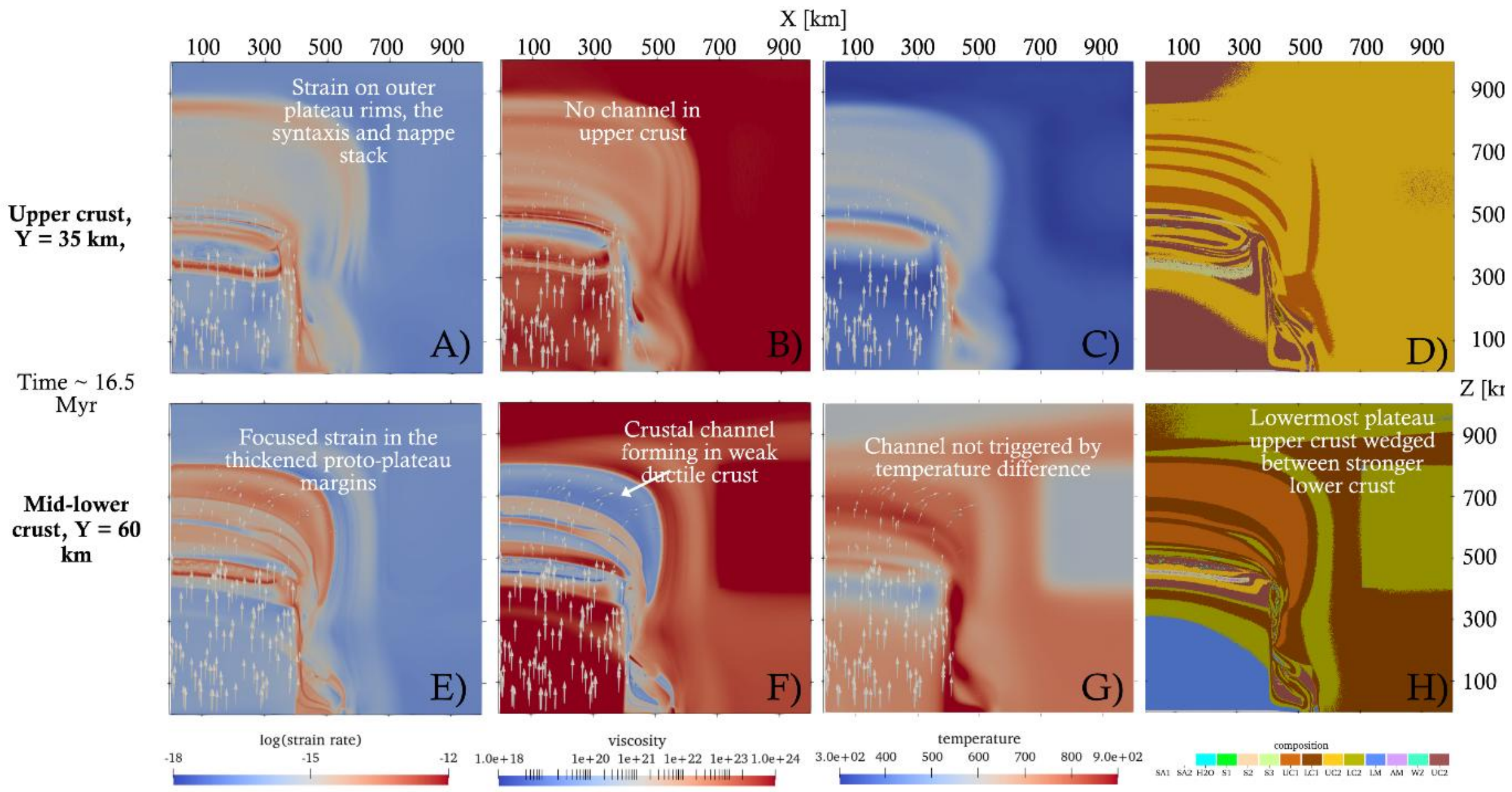

Figure S1 | Decoupling between the upper and lower crust during channel formation in the mid-lower crust in the reference model. The horizontal velocity field is depicted as solid white arrow glyphs on top of the strain rate (A, E) effective viscosity (in Pa s, panels B, F) and temperature (in °K, panels C, G) for upper (top row) and mid-lower (bottom row) crustal depth slices. The composition is shown in panels D and H, respectively. The viscosity slices show that the flow localises in a narrow channel (and not as a "broad flow"). This is because the weak mid-lower crust is wedged between stronger, lower crustal units (composition panel on the right) in response to northward advance of the indenter.

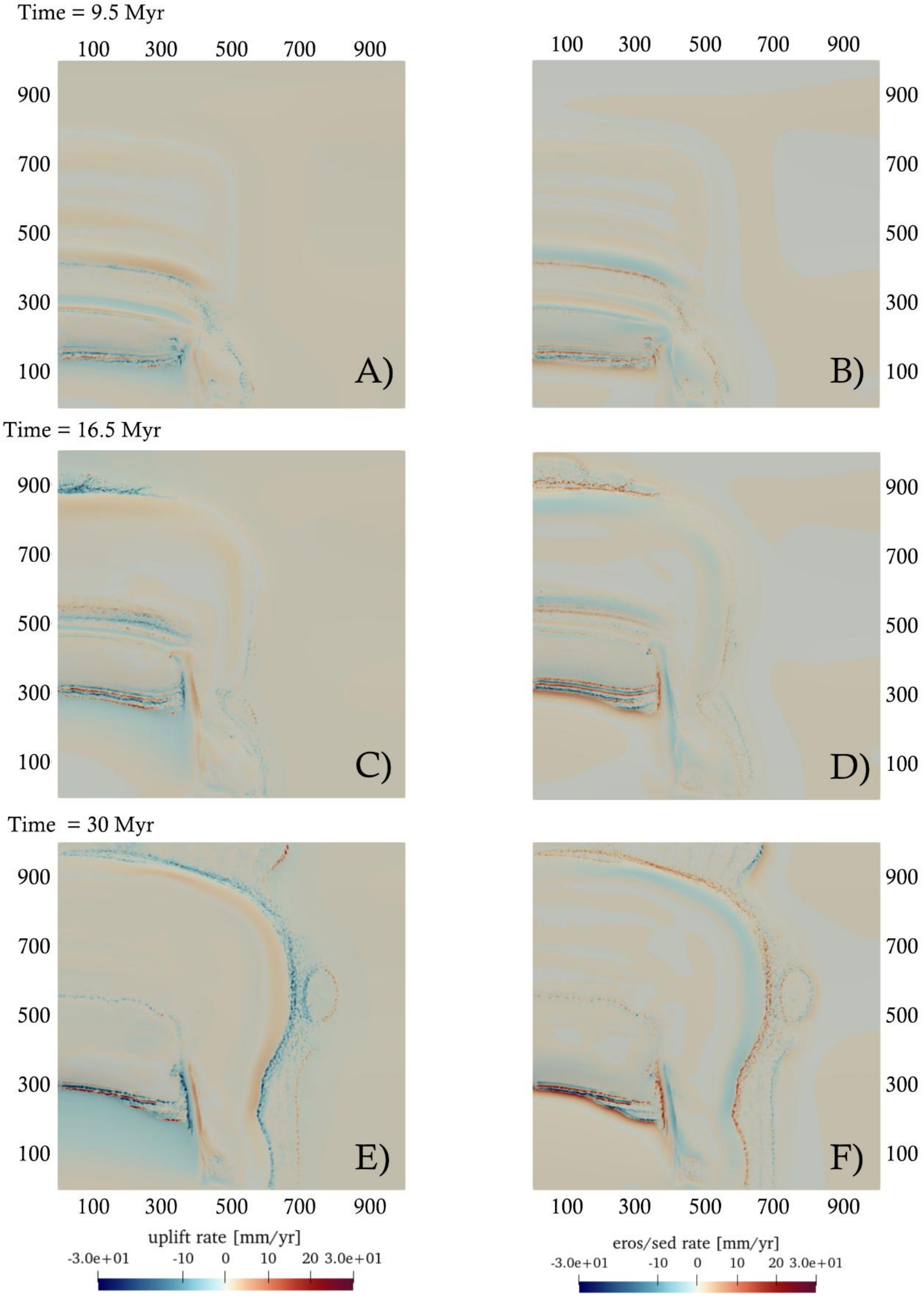

Figure S2 | uplift/subsidence and erosion/sedimentation rates before, during and after syntaxis formation in our reference model. The rates have been converted to mm/yr. The highest rates are in active faults in the syntaxis, main orogen and outer rim of the plateau.

The influence of surface processes

We tested different diffusion coefficients to simulate different erosion and sedimentation rates (see Methods). Increasing this coefficient from 500 (reference model parameter in Fig. S2) to 5000 m^2/yr (Fig. S3) leads to ~7 mm/yr erosion in the core of the Himalayas, equivalent to removing the entire upper and lower crust within the first 5 Myr. Such a high diffusion coefficient still leads to the development of a syntaxis and ductile mid-lower crustal channel (Figure S3). In the orogenic core of the reference model, the erosion rate in the first 7 Myr is ~3.2 mm/yr (Figure S2). Enhanced erosion leads to a weaker crustal flow behind the syntaxis (Figure S3). This is because there is no indentation pulse at mid-lower crustal and mantle depths that is observed in the reference model, hence the peak channel flow velocities are lower (Figure S4). The crustal channel shallows in its eastern segment by about 10 km before stagnating by ~ 25 Myr (Figure S4). In this simulation the syntaxis is relatively narrow and small until ~20 Myr. The syntaxis is coupled to the rest of the orogen, as it is not constrained by strike-slip faults along its flanks. The syntaxis position initially follows the pattern of the reference model until ~ 18 Myr, advancing northward slightly faster than the main orogenic front (Figure S3). Between 18 and 30 Myr numerical time, the syntaxis moves ~100 km eastward, changing in strike from N-S to NE-SW. The syntaxis clearly follows the expansion of the orogenic plateau without relative offsets due to strike-slip faults, suggesting that erosion intensity has a strong control on the deformation mechanisms in the syntaxis area.

The topographic evolution with less efficient surface processes on first order resembles the reference model, showing a slightly higher orogen in front of a slightly smaller plateau (Fig. S5). The topography remains high throughout the simulation as erosion does not keep pace with crustal thickening. The crustal thickness in the plateau reaches 100 km where the indenter crust underthrusts.The Indian slab advances much further than in the reference model. The syntaxis itself initially follows a similar evolution and formation as the reference model: the thrusts of the nappe stack of the main orogen connect through an oblique thrust with the plate boundary between the indenter and the Eurasian margin, forming a strike-slip fault zone. The corner then tears apart along this right-lateral strike-slip fault. The Indian upper crust in the corner folds into a proto-syntaxis, which starts to elongate with increasing slip along the strike-slip fault. The folding is less tight as in the reference model due to less efficient erosional unroofing.

The erosion rates in the Namche Barwa syntaxis were 4-7 mm/yr for the past 3 Myr (Bracciali et al. 2016), matching our modelled erosion rates reasonably well. The short-term erosion rates in the syntaxis in our reference model are up to 10 mm/yr along active shear zones and 0-2 mm/yr elsewhere (Fig. S1), matching reported rates of 0.1-4 mm/yr (Finnegan et al. 2008).

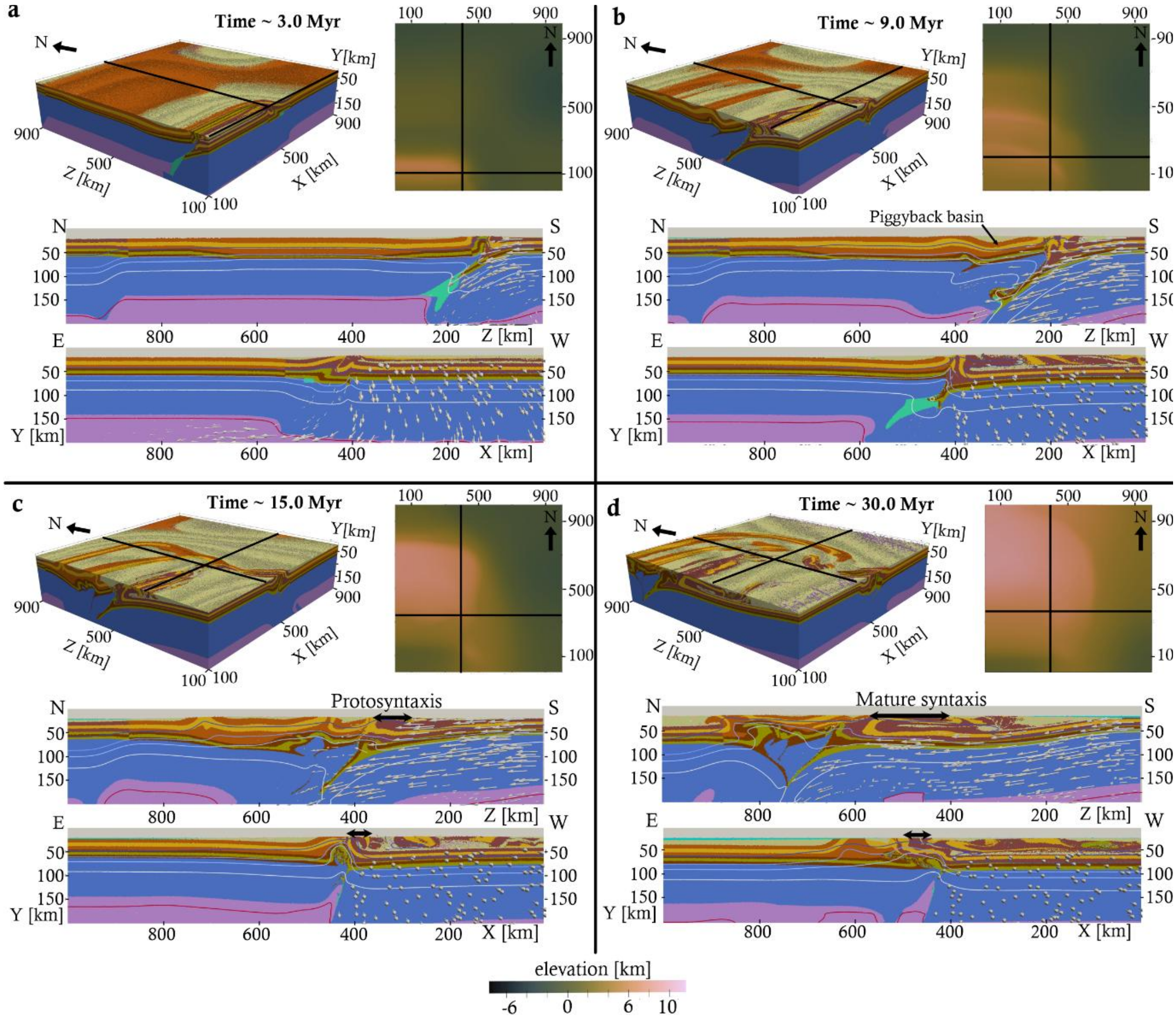

Figure S3 | Syntaxis development under a more efficient surface process conditions. The surface processes diffusion coefficient is increased by an order of magnitude when compared with the reference model. Model is shown by rock composition 3D view, cross-sections and topography results. There are major differences in the structure of the pro-wedge and localisation of strike-slip faults. Nevertheless, the main patterns hold and a syntaxis still forms. The final crustal thickness of the plateau is ~20 km lower than in the reference model.

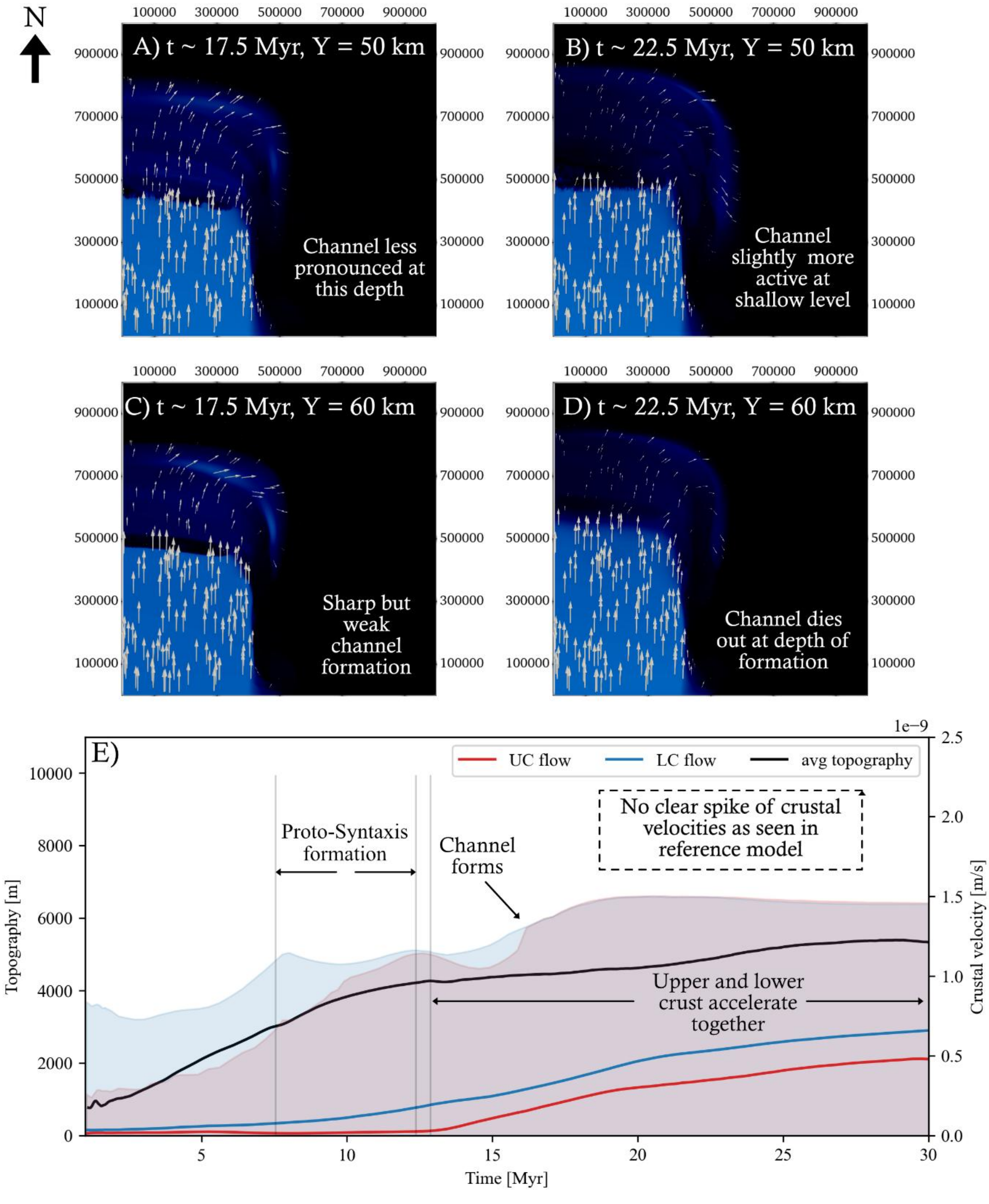

Figure S4 | Channel shallowing and syntaxis uplift vs crustal flow for strong erosion model. A-D) Horizontal depth slices at Y = 50 and Y = 60 km showing the horizontal velocity similar to Fig. 3 in the main text. E) Same plot as in Extended Data Figure 5 for the reference model, but for the high erosion model. The most notable differences are a lower final topography, and the lower crustal velocities after channel formation.

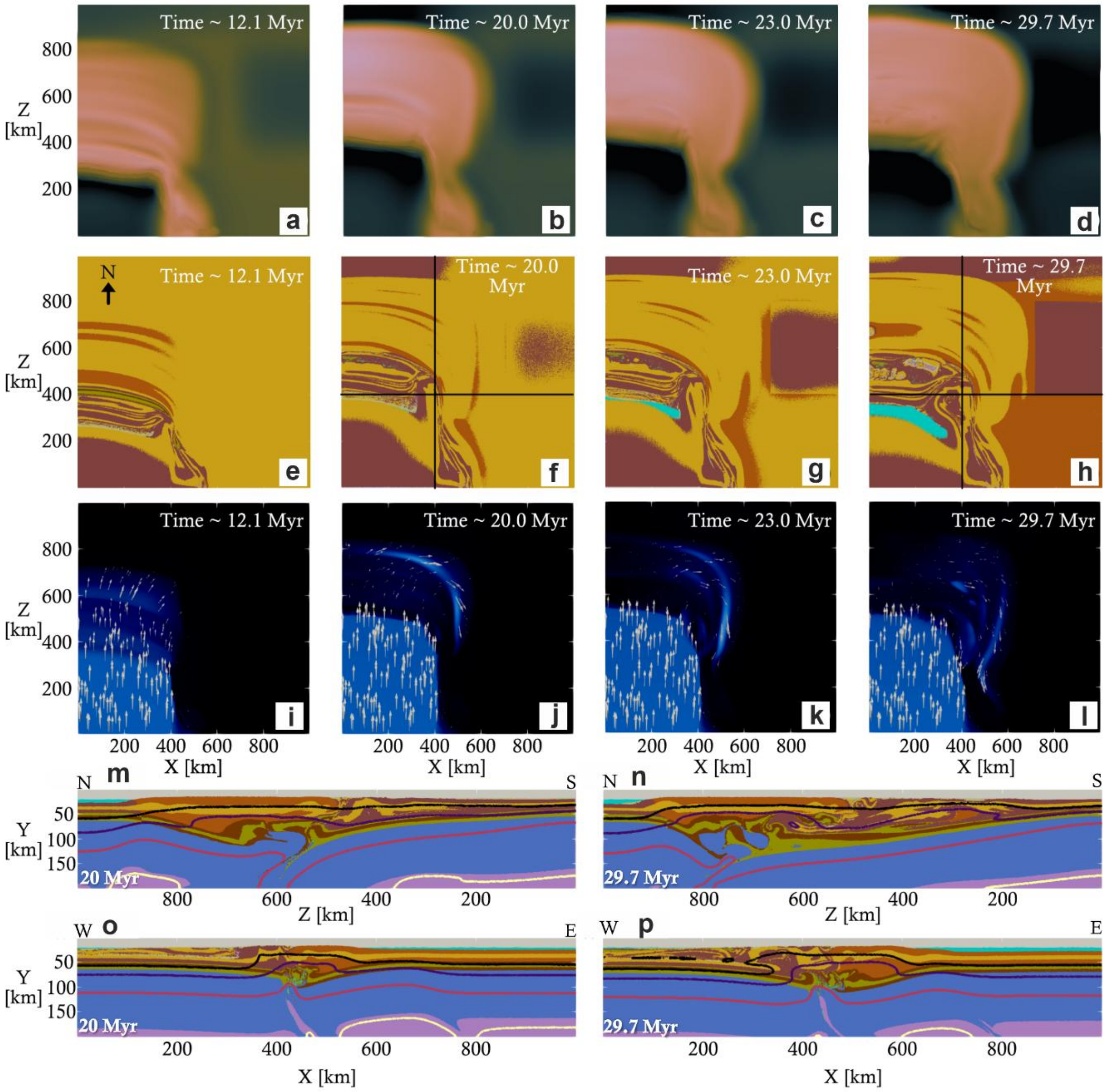

Figure S5 | Syntaxis development under inefficient surface process conditions. The surface processes diffusion coefficient is decreased by an order of magnitude when compared with the reference model. Model is shown by topography (a-d), rock composition horizontal slices (e-h), ductile crustal velocity field (i-l) and rock composition cross-sections (m-p). Same topography scale is applied as in Extended Data Figure 9.

References

Bracciali, L. *et al.* Plio-Pleistocene exhumation of the eastern Himalayan syntaxis and its domal 'pop-up'. *Earth-Sci. Rev.* **160**, 350–385 (2016).

Finnegan, N. J. *et al.* Coupling of rock uplift and river incision in the Namche Barwa-Gyala Peri massif, Tibet. *Geol. Soc. Am. Bull.* **120**, 142–155 (2008).
